# Coordinated voltage control in renewable energy integrated power systems using ant colony optimization

**DOI:** 10.1038/s41598-026-54302-9

**Published:** 2026-06-08

**Authors:** K. Durgadevi, A. Murugesan, Viharika Chaudhari, Yagnik Rathod

**Affiliations:** 1 ECE Department, SRM Valliammai Engineering College, Chennai, India; 2 Department of Biomedical Engineering, K.S.R. College of Engineering, Tiruchengode, Namakkal [District], Tamil Nadu India; 3 Computer Engineering Department, Government Engineering College, Dahod, India

**Keywords:** Renewable energy integration, Voltage stability, Ant colony optimization, Deep Q-networks, Sustainable energy, Grid optimization and grid resilience, Energy science and technology, Engineering

## Abstract

Navigating the complexities of modern power systems with a high share of renewable energy sources (RES) requires effective control strategies. This research presents a new Coordinated Voltage Control (CVC) framework for RES-integrated grids by combining Ant Colony Optimization (ACO) with a Deep Q-Network (DQN) controller. In this hybrid approach, ACO optimizes voltage profiles while DQN adjusts reactive power support in real-time, allowing for quick responses to grid conditions. The proposed method significantly improves voltage stability and reduces system losses, showing strong performance under different renewable generation and load scenarios. The ACO and DQN framework also shows good computational efficiency, achieving convergence in about 45 to 50 iterations. By using historical and simulated data for DQN training, the controller predicts the best reactive power actions, ensuring scalable and reliable voltage regulation. This work provides a practical and flexible solution for modern power systems rich in renewables, supporting better grid stability and operational efficiency.

## Introduction

**Fig. 1:**
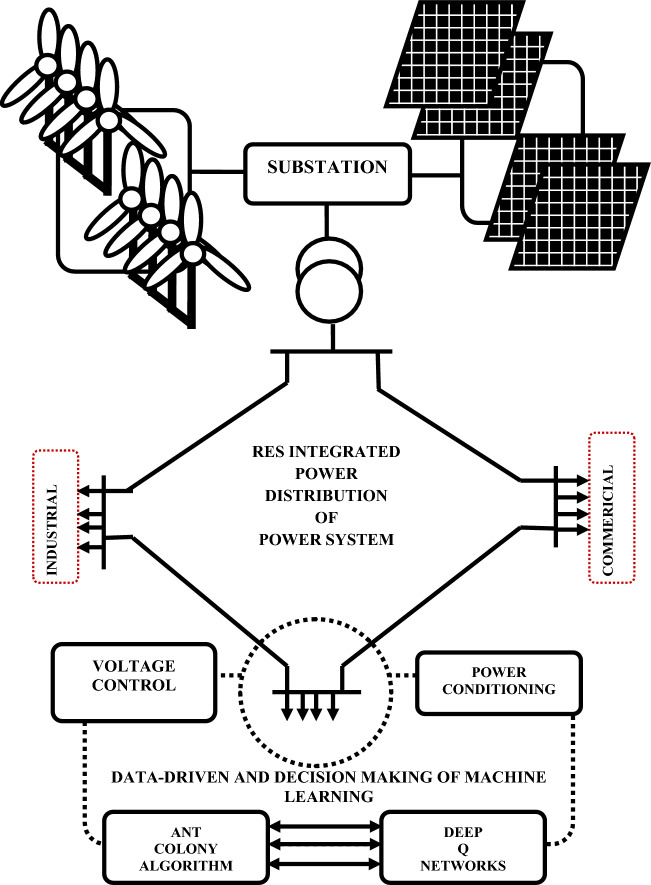
Voltage control RES integrated power system by machine learning.

The increasing penetration of RES such as wind and solar power introduces significant challenges in maintaining voltage stability due to their intermittent and variable nature^1^. This research unveils a pioneering study on Coordinated Voltage Control (CVC) in renewable energy integrated grids, harnessing the power of Ant Colony Optimization (ACO) and a Deep Q-Network (DQN) based controller^2,3^. The proposed ACO-DQN-based coordinated framework of the voltage control presents the functional structure of the coordinated system and the interaction between the system components, as shown in Fig. 1.

ACO fine-tunes voltage profiles while DQN dynamically adjusts reactive power support, ensuring real-time adaptability to grid conditions. The proposed methodology not only fortifies voltage stability but also underscores scalability and adaptability in managing intricate power systems^4,5^.

The general design of the desired voltage control system of a renewable energy integrated power system is depicted in Fig. 1. The machine learning layer of data collection is a collection of real-time measurements like the magnitude of voltage, power flowing, and levels of the renewable generation. Ant Colony Optimization (ACO) module is an optimization method used to implement global voltage profile optimization whereby it identifies the optimum control settings of the voltage regulators and reactive power devices. Deep Q-Network (DQN) controller is a dynamic control of reactive power support, depending on the system states and the learned policies. A combination of these elements allows the industry, commercial and residential loads to be coordinated and adapted to control their voltage at all times.

Although the integration of renewable energy has seen much advancement, the issue of voltage stability in the distribution networks where the renewable energy sources are in large numbers (Distribution of renewable energy and its effects on utility networks, 2013) is a notable challenge. The highly discontinuous and intermittent characteristics of wind and solar generation cause system losses to be highly discontinuous, as well as the power quality and intermittency to be high. The current voltage control methods tend to be centralized, non-portable, or device-specific and are therefore unable to deal with uncertainties that happen at the state of real-time, large-scale applications, and coordination of many voltage regulation devices. As such, it is apparent that an adaptive, scaled and coordinated voltage control scheme that can respond to dynamic operating conditions in RES-integrated power systems is necessary.

### Background and motivation

This research is motivated by the fact that the nature of voltage regulation of renewable rich power systems is becoming increasingly complicated. Conventional voltage control systems are not smart enough to control distributed and highly varying power sources. Recent optimization and reinforcement learning, make it possible to create adaptive control strategies that will be able to cope with these challenges. The proposed research is driven by the necessity to reduce the existing divide between offline optimization and online decision-making using a coordinated ACO-DQN system.

### Objectives and scope of this research

At the core of this research lies the ambition to engineer a cutting-edge Coordinated Voltage Control (CVC) strategy meticulously crafted to suit the intricacies of renewable energy integrated power systems^6^. This strategy stands on the shoulders of two pivotal pillars: Ant Colony Optimization (ACO) and a Deep Q-Network (DQN) based controller. ACO acts as the guiding force behind the optimization of voltage profiles, meticulously sculpting them to minimize deviations and uphold stable voltage levels across the power network. Concurrently, the DQN controller assumes the role of an adaptive sentinel, dynamically modulating reactive power support in response to the ever-shifting landscape of real-time grid conditions^7^. This symbiotic fusion promises not only enhanced adaptability but also fortified stability within the power system’s framework. With a laser focus on achieving tangible outcomes, the research outlines four specific objectives:

To achieve the objectives of this research, a novel approach is employed, combining Ant Colony Optimization (ACO) and a Deep Q-Network (DQN) based controller for coordinated voltage control in renewable energy integrated power systems.

### Ant colony optimization (ACO)

ACO is a nature-inspired metaheuristic algorithm that emulates the foraging behavior of ants to find optimal solutions. In this context, ACO is utilized to optimize voltage profiles and minimize voltage deviations across the power network^8^. The algorithm iteratively explores potential paths for voltage control, simulating the pheromone-laying and following behavior of ants to identify the most efficient solutions.

### Deep Q-network (DQN) controller

DQN is a reinforcement learning algorithm that combines Q-learning with deep neural networks. In this research, the DQN controller is specifically designed to dynamically adjust reactive power support based on real-time grid conditions^9^. Through training on historical and simulated data, the DQN controller learns to predict optimal actions for reactive power adjustment, ensuring robust performance under varying grid conditions.

### Integration of ACO and DQN

The proposed Coordinated Voltage Control (CVC) strategy involves the seamless integration of ACO and DQN to enhance voltage control capabilities. While ACO focuses on optimizing voltage profiles across the network, the DQN controller dynamically adjusts reactive power support, providing a comprehensive and adaptive solution for voltage stability in renewable energy integrated power systems^10^. This integration enables efficient and effective control of voltages to meet the strong and formidable challenge of the variability of the sources of renewable energy as well as to guarantee the stability and reliability of the power system. Simultaneously, this integration attempts to navigate through the overall lifecycle of the proposed CVC strategy—starting with its inception to its concrete implementation and subsequent evaluation^11^. This holistic method involves carrying out testing of the strategy under intensive experimentation through a standardized test system that would be combined with wind and solar power stations. Moreover, the study also ventures further on the assimilation of other renewable energy and the latest grid technologies, therefore extending the frontiers of innovation in order to deliver the next generation of strength and effectiveness in the power system environment.

**Fig. 2:**
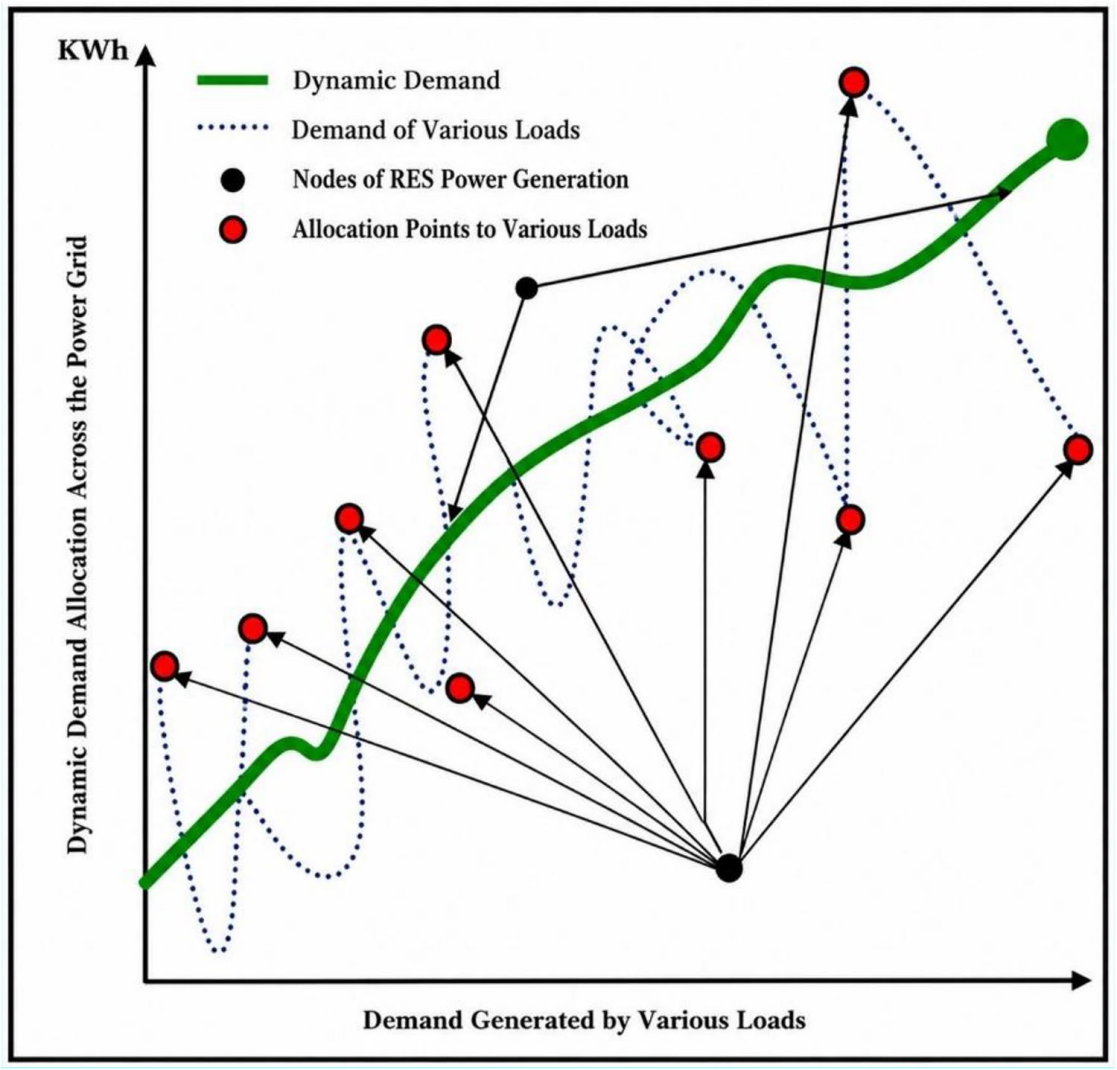
Adaptive voltage management for coordinated renewable energy integrated power systems.

Figure 2 indicates how the voltages are distributed among the various buses in wind pv integrated distribution system.

### Proposed methodologies

In pursuit of its objectives, this research employs a sophisticated amalgamation of Ant Colony Optimization (ACO) and a Deep Q-Network (DQN) based controller to orchestrate coordinated voltage control within renewable energy integrated power systems. ACO, inspired by the foraging behavior of ants, serves as the cornerstone of this strategy^12^. This nature-inspired metaheuristic algorithm operates by simulating the intricate dance of pheromone-laying and following exhibited by ant colonies. In the context of voltage control, ACO meticulously navigates the labyrinth of power networks, iteratively seeking optimal solutions to optimize voltage profiles and minimize deviations. By emulating the collective intelligence of ants, ACO identifies the most efficient pathways for voltage control, thereby bolstering the stability and reliability of the power system. Complementing ACO’s prowess is the formidable capabilities of the Deep Q-Network (DQN) controller, a reinforcement learning algorithm fortified by the fusion of Q-learning and deep neural networks^13^. Designed to dynamically adjust reactive power support in response to real-time grid conditions, the DQN controller emerges as a stalwart guardian of system stability. Trained using a wealth of historical and simulated data, the DQN controller harnesses the power of machine learning to predict optimal actions for reactive power adjustment, ensuring steadfast performance under the ever-fluctuating landscape of varying grid conditions. The true synergy of this strategy lies in the seamless integration of ACO and DQN, each complementing the other’s strengths to forge a comprehensive solution for voltage stability. While ACO meticulously optimizes voltage profiles, DQN dynamically fine-tunes reactive power support, collectively fortifying the system’s resilience in the face of renewable energy integration. This integration not only enhances voltage control capabilities but also heralds a new era of adaptability and efficiency within renewable energy integrated power systems, laying the groundwork for a sustainable and robust energy future^14,15^.

### Experimental validation and results

The proposed Coordinated Voltage Control (CVC) strategy undergoes rigorous evaluation using a standardized test system that incorporates both wind and solar power plants. This evaluation process employs a battery of key performance metrics to gauge the effectiveness and efficiency of the ACO-based CVC strategy, shedding light on its impact on voltage stability and system efficiency^16^. Voltage deviation serves as a pivotal indicator of the strategy’s efficacy in maintaining stable voltage levels across the power network. The implementation of the ACO-based CVC strategy yields notable reductions in voltage deviation, showcasing its ability to mitigate fluctuations and uphold a consistent voltage profile, thus bolstering overall system stability. In addition to mitigating voltage deviation, the ACO-based CVC strategy also demonstrates prowess in reducing system losses. By optimizing voltage profiles and fine-tuning reactive power support, the strategy contributes to enhance system efficiency, thereby minimizing losses and improving overall grid performance^17^. The computational efficiency of the ACO algorithm is another critical aspect evaluated during the assessment of the CVC strategy. Rapid convergence of the algorithm ensures timely voltage control actions, facilitating swift responses to fluctuations and disturbances within the power network, thereby enhancing system reliability. Furthermore, the evaluation delves into the integration of the DQN-based controller, which augments the capabilities of the CVC strategy Although convergence time has been slightly extended by this complexity added to the control scheme, the predictive capabilities of the DQN controller allow even further shortening of the deviation of the voltage and system losses. This highlights its contribution to a healthy performance in different grid conditions thereby enhancing the strength of the power system when it comes to dynamic operation issues. Collectively, the evaluation of the CVC strategy provides compelling evidence of its efficacy in enhancing voltage stability and system efficiency within renewable energy integrated power systems^18^. The integration of advanced control techniques such as ACO and DQN heralds a new era of adaptive and resilient grid management, paving the way for a sustainable and efficient energy future. This research marks a significant advancement in voltage control for renewable energy integrated power systems. By combining Ant Colony Optimization and a Deep Q-Network based controller, the proposed coordinated voltage control strategy effectively addresses the challenges associated with renewable energy integration^19^. The results demonstrate substantial improvements in voltage stability and system efficiency, paving the way for resilient energy infrastructure and transformative advancements in grid optimization. Future work will explore real-time implementation and the integration of additional renewable sources and advanced grid technologies, ensuring continued improvements in voltage stability and system efficiency^20,21^.

More recent papers have dealt with the issue of voltage regulation in renewable energy source (RES)-integrated power systems with recent optimization and intelligent control methods^22–27^. Proactive voltage regulation schemes on bio-inspired optimization and coordinated distributed energy resource control have shown the capability of reducing voltage profiles better, but frequently depend on centralized or offline optimization^22,24^. Reviews have noted the increasing complexity of simultaneous voltage and frequency regulation with large renewable penetration levels and the necessity to use hybrid learning-optimization solutions^23,26^. Active power curtailment techniques and the use of smart inverter based methods of voltage control can be successfully used to limit the problem of overvoltage but they can also contribute to loss of renewable energy and reduced adaptability^25^. The optimization of Ant Colony has performed well in tackling nonlinear and combinatorial optimization problems in power systems including energy storage, but its usage in coordinated voltage control in the real-time has been not well explored^27^. These shortcomings are driving the creation of a hybrid ACO-DQN model that can dynamically control the voltage in real-time to accommodate real-time changes in renewable and load.

Improvement techniques have been applied to energy harvesting systems connected to the grid. These methods show better operational efficiency and system reliability^28^. Deep reinforcement learning has been used to create optimized controllers for renewable energy systems. This helps with voltage and power management under changing conditions^29,30^. In addition, demand-side control strategies that use deep learning optimization have achieved effective energy savings and better coordination in renewable-integrated networks^31^. However, most approaches depend on offline optimization or lack real-time adaptability. This reveals the need for hybrid frameworks that mix metaheuristic optimization with reinforcement learning, such as the proposed ACO-DQN strategy.

The performance of the experimental validation of the proposed ACODQN coordinated voltage control framework was conducted based on comparison with other recent advances in optimization of the intelligent power systems and integration of renewable energy. The concept of adaptive and intelligent control strategies in the present-day smart grid communication and demand response mechanisms underline the necessity of a stable operation in the conditions of dynamic loads^32^. Moreover, load profile reconstruction using machine learning methods has also greatly enhanced the trustworthiness of power system data to be used in training more advanced predictive and reinforcement learning models^33^.

As the electric vehicles become a booming industry, the optimal planning strategy of the electric charging infrastructure has also become critical in ensuring that the grid does not collapse and that the energy is used efficiently^34^. Power electronic interfaces modeling is very important in testing sophisticated grid control frameworks. The effective modelling methods of modular multilevel converters can enhance accuracy in the simulation and provide realistic testing of the coordinated control method at renewable energy systems^35^. On the same note, higher modulation methods in dual-active-bridge converters increase energy conversion in distributed power systems^36^. More sophisticated control of neutral-point-clamped converters also allows stable operation of conversion of power under changing load conditions^37^.

The design and operation of power system components are also optimized, which leads to the increased system performance. Multi-objective optimization tools assisted by surrogate models can be effectively used to accomplish design enhancing electrical machines in systems based on renewable sources^38^. Multiparallel converter architectures operating in a fault tolerant mode lead to better resilience of the system and reliability of operation in more complex grid environments^39^. Also, the works of research that examined the effect of unbalanced inductance on PWM setups illustrate the sensitivity of converter models to ensure a stable operation^40^. The strategies of energy management are also important in the validation of intelligent grid control strategies. Scheduling of demand response and energy storage can greatly enhance the economic dispatch of micro grids based on wind integration^41^. The use of carbon capture technologies in low-carbon optimization strategies will also facilitate sustainability in the operation of power systems^42^. Further, data-driven technologies and uncertainty beneficial optimal power flow schemes can be used as effective methods of stability in the voltage of active distribution systems^43^,^44^.

The recent developments in the disturbance monitoring and system planning also lead to better grid stability. The voltage sag methods are useful in detection of perturbation in power system rich in renewable sources^45^. Large-scale unit commitment optimization based on data methods also improve the effectiveness of working with systems^46^. Synchronous condensers are designed to operate in renewable power systems with high voltage support by optimization of design^47^. The high technology of DC transformers is used to enable the economy of integration of photovoltaic plants^48^. Multi-energy systems Hydrogen energy storage plans also offer extra flexibility^49^. Lastly, the use of voltage stability support methods to wind farm integrated HVDC systems illustrates the significance of coordinated control methods in the high renewable penetration circumstances^50^.

Coordinated Voltage Control (CVC) strategy has been suggested and run and tested with MATLAB/Simulink R2023a coupled to MATPOWER to analyze power flow. The test platform was a modified IEEE XX-bus distribution test system with inbuilt wind and photovoltaic (PV) generation units. The simulations were implemented in an Intel i7 processor, 16 GB RAM based system. The degree of penetration of renewable energy was set at 30 percent, 70 percent, to test the resiliency of the system in various operating conditions. The optimization of offline voltage profile had been performed with the help of the Ant Colony Optimization (ACO) algorithm, whereas the Deep Q-Network (DQN) controller had been trained and implemented to perform real-time reactive power control.

### Research gap and contribution

Although several studies have looked into voltage control in renewable energy integrated power systems using heuristic optimization and machine learning techniques, most current approaches struggle with limited adaptability, a lack of coordinated control, and insufficient real-time learning ability. Many methods depend on static optimization or single-algorithm solutions, which cannot respond effectively to rapid changes in renewable generation. To overcome these issues, this research suggests a hybrid ACO and DQN framework that merges global optimization with real-time reinforcement learning. This combination allows for coordinated, adaptable, and scalable voltage control in renewable energy integrated power systems.

## Enhancing voltage stability through renewable energy integration

Renewable energy sources (RES) integration with the current power systems is a radically different approach to sustainable energy generation. Nonetheless, this shift poses major challenges, especially on the aspect of voltage stability^1^. As compared to conventional sources of power generation, renewable sources like solar and wind are automatically considered as variation and intermittence of power generation, thereby causing power output variation that disrupts the voltage stability level in the grid. This part will discuss the particular issues surrounding the concept of voltage stability when it comes to integrating renewable energy sources and the major deliberations that can be made in order to deal with such issues. The intermittency of renewable energy sources is the unpredictability and variability of power production^2^. Meanwhile, whereas the fossil fuel power plants can run 24/7 and are predictable, renewable energy sources such as solar photovoltaic (PV) systems and wind turbines are dependent on natural forces e.g. sunlight and wind to produce electricity.

For instance, solar PV systems generate electricity only when exposed to sunlight, meaning they are inactive during nighttime or when obstructed by clouds. Similarly, wind turbines produce power based on the availability and strength of wind, which can vary significantly depending on weather conditions and geographical location^3^. These fluctuations in renewable energy generation can lead to rapid changes in power output within the grid. When renewable energy generation suddenly increases due to a gust of wind or a clearing in the clouds, the excess power injected into the grid can cause voltage levels to rise abruptly. Conversely, when renewable energy generation decreases, such as during periods of low sunlight or wind, voltage levels may drop, potentially leading to voltage instability or even voltage collapse. As the penetration of renewable energy sources continues to grow, managing these fluctuations becomes increasingly challenging^4^. Without proper coordination and control mechanisms in place, the intermittent nature of renewable energy sources can destabilize the grid, compromising its stability and reliability. Hence, the management and reduction of the effects of the intermittency of the Renewable energy patterns is paramount in terms of ensuring stability and resilience of the power system at large^5^. The Renewable Energy Generation can be said to be as follows:

1 $${P}_{RES}^{Control}={A}_{Solar-Wind}^{Control}\times {G}_{Solar-Wind}^{Control}\times {\eta}_{Solar-Wind}^{Control}$$

where: ${P}_{RES}^{Control}$​ is the Renewable energy generation, ${A}_{Solar-Wind}^{Control}$ is the Area of solar panels or wind turbine blades ${G}_{Solar-Wind}^{Control}$ is the Solar irradiance or wind speed, ${\eta}_{Solar-Wind}^{Control}$. Efficiency of the solar panels or wind turbine^1^. The variability in power output refers to the fluctuations or changes in the amount of electricity generated by renewable energy sources over time^2^.

Renewable energy sources like solar PV systems and wind turbines are variable unlike the conventional power plants that can operate with a relatively stable output because of a number of factors such as weather patterns, time of the day and season changes. The weather conditions are important in the establishment of the power output of renewable energy sources^3^. For example, solar PV systems rely on sunlight to generate electricity, and variations in cloud cover can cause rapid fluctuations in solar irradiance, leading to corresponding changes in power output. The voltage stability index (VSI) is expressed as:

2 $${VSI}_{Control}^{RES}=\frac{{V}_{Max}^{RES}-{V}_{Min}^{RES}}{{V}_{nom}^{RES}}\times 100$$

where $${\mathrm{V}}_{\mathrm{Max}}^{\mathrm{RES}}$$ is the Maximum voltage level, $${\mathrm{V}}_{\mathrm{Min}}^{\mathrm{RES}}$$ is a Minimum voltage level, $${\mathrm{V}}_{\mathrm{nom}}^{\mathrm{RES}}$$ is the Nominal voltage level. Similarly, wind turbines are sensitive to wind speed and direction, and shifts in atmospheric conditions can result in fluctuations in wind power generation^4^. The power output of renewable energy sources can vary throughout the day due to diurnal patterns in solar radiation and wind speed. Solar PV systems typically generate the highest output during daylight hours when solar irradiance is at its peak, while wind turbines may experience increased power generation during certain times of the day when wind speeds are higher. Seasonal variations in weather patterns can also influence the power output of renewable energy sources^5^. For instance, solar PV systems may produce more electricity during the summer months when days are longer and sunlight intensity is higher, while wind turbines may experience increased generation during certain seasons characterized by stronger winds. The real ($${P}_{Real}^{RES})$$ and Reactive powers ($${Q}_{Real}^{RES})$$ of RES integrated power system can be expressed as:

3 $${P}_{Real}^{RES}={V}_{PS}^{RES}\times {I}_{PS}^{RES}\times Cos(\theta )$$

and

4 $${Q}_{Real}^{RES}={V}_{PS}^{RES}\times {I}_{PS}^{RES}\times Sin(\theta )$$

where ${P}_{Real}^{RES}$ is the Active power, ${Q}_{Real}^{RES}$ is the reactive power ${V}_{PS}^{RES}$ is the Voltage magnitude, ${I}_{PS}^{RES}$ is Current magnitude, *θ* is Phase angle difference^51^. Managing the variability in renewable energy generation requires sophisticated control strategies to maintain voltage stability and prevent voltage excursions beyond acceptable limits. Utilities and grid operators employ advanced forecasting techniques to predict renewable energy generation patterns and adjust grid operations accordingly^52^. The Voltage deviations can be calculated by:

5 $${V}_{Dev}^{RES}={V}_{Act}^{RES}-{V}_{Target}^{RES}$$

where ${V}_{Act}^{RES}$ is the Actual Voltage Level, ${V}_{Target}^{RES}$ is the Target voltage level. Also, grid connected energy storage systems and flexible demand-side management program can be used to address the effects of the variability of renewable energy by offering grid balancing services and could maximise the exploitation of the renewable energy resources^1^. In general, it is necessary to efficiently deal with changes in the renewable energy production to guarantee the stability and reliability of the power grid and manage this problem with the combination of sophisticated control options, foreseeing methods, and grid flexibility options^2^. DERs are a wide array of decentralized sources of energy such as rooftop panels or small wind turbines, energy storage systems, all of which are linked to the points of the power grid at a distribution level that are nodes on the voltage.

While DERs offer numerous benefits such as increased energy efficiency, reduced emissions, and enhanced grid resilience, their integration also introduces significant challenges, particularly concerning voltage stability. Unlike centralized power generation facilities, which are typically connected to the transmission-level voltage nodes of the grid, DERs are directly interconnected with the distribution network^3^. This distribution-level connection places DERs closer to end-users and enables more localized energy generation and consumption. However, it also means that DERs have a direct impact on distribution-level voltage levels, leading to localized voltage variations within the grid. The proliferation of DERs can result in localized voltage fluctuations, which can manifest as overvoltage or undervoltage conditions at distribution-level voltage nodes. For example, during periods of high solar generation, voltage levels may rise due to excess power injection from rooftop solar panels, leading to overvoltage issues^4^. Conversely, when DERs are not generating sufficient power, voltage levels may drop, potentially causing undervoltage conditions. Coordinating voltage control across multiple DERs while ensuring overall grid stability presents a significant technical and operational challenge for utilities and grid operators. Unlike traditional centralized generation, which can be controlled and coordinated from a single point, DERs are dispersed across the grid and operated by individual owners or operators^5^. To deal with the issue of voltage stability in this distributed environment, complex control measures and coordination techniques are needed to guarantee that the levels of voltage remain within reasonable levels. To deal with the challenges of DERs on the management of voltage stability, a holistic treatment is required that incorporates advanced control algorithms, real time monitoring and controls systems, as well as grid-edge intelligence^51^. Both utilities and grid operators will need to formulate and execute strategies to be active on the management of those voltages changes by DERs without bringing any problem to the overall grid stability and reliability^1^. It can be the implementation of modern-voltage control equipment, including voltage regulators and capacitor banks, and the use of data analytics and communication technologies to use the voltage control in real-time. Moreover, stakeholders, such as DER owners, grid operators and regulatory authorities must work together and coordinate its activities so that the difficulties linked to the integration of DER could be appropriately dealt with to guarantee the stability and resilience of the power grid. The proposed Coordinated Voltage Control (CVC) strategy undergoes rigorous evaluation using a standardized test system that incorporates both wind and solar power plants. This evaluation process employs a battery of key performance metrics to gauge the effectiveness and efficiency of the ACO-based CVC strategy, shedding light on its impact on voltage stability and system efficiency^2^. Voltage deviation serves as a pivotal indicator of the strategy’s efficacy in maintaining stable voltage levels across the power network. The implementation of the ACO-based CVC strategy yields notable reductions in voltage deviation, showcasing its ability to mitigate fluctuations and uphold a consistent voltage profile, thus bolstering overall system stability. In addition to mitigating voltage deviation, the ACO-based CVC strategy also demonstrates prowess in reducing system losses. By optimizing voltage profiles and fine-tuning reactive power support, the strategy contributes to enhance system efficiency, thereby minimizing losses and improving overall grid performance^3^. The computational efficiency of the ACO algorithm is another critical aspect evaluated during the assessment of the CVC strategy. Rapid convergence of the algorithm ensures timely voltage control actions, facilitating swift responses to fluctuations and disturbances within the power network, thereby enhancing system reliability. Furthermore, the evaluation delves into the integration of the DQN-based controller, which augments the capabilities of the CVC strategy. Despite a slight increase in convergence time attributable to the added complexity of the control scheme, the DQN controller’s predictive prowess enables further reductions in voltage deviation and system losses^4^. The analysis of the CVC strategy, taken as a whole, offers strong indications of its effectiveness in strengthening the stability of voltages and system performance in operational conditions subject to dynamical changes in renewable energy integrated power systems. Future grid management with the incorporation of smart control models like ACO and DQN is a sign of a new grid that can modify itself and stay resilient, which will lead to the future of energy that is sustainable and efficient^5,51^.

### Voltage profile analysis of distribution buses

The voltage profile of selected buses prior to and after the adoption of the proposed ACO DQN-based CVC strategy is shown in Table 1. Before control, the voltage violations of multiple buses were found to be more than a percentage of over 5 times because of the high wind-PV penetration. Following the implementation, voltages on all buses were maintained within reasonable limits which proved the efficiency of coordinated control.

**Table 1:** Bus voltage profile comparison.

Bus no	Before control (p.u.)	After ACO–DQN (p.u.)
5	0.93	0.98
12	0.91	0.97
18	1.06	1.01
25	0.92	0.99

### Need for coordinated voltage control strategies

**Fig. 3:**
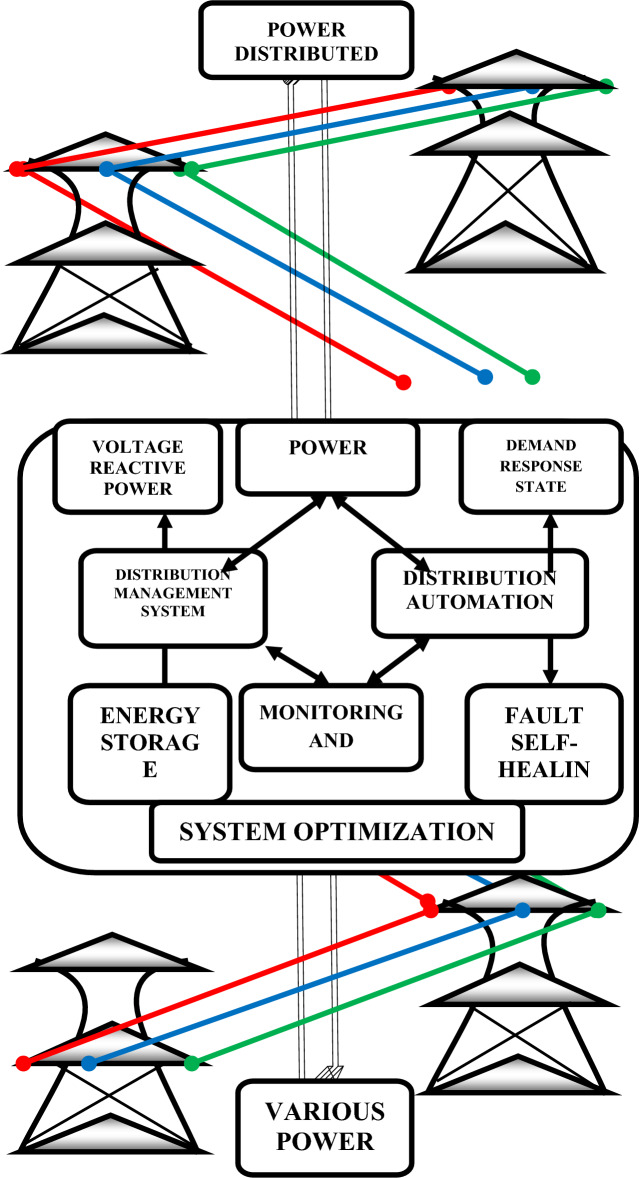
Scenario for power forecasting for optimal voltage monitoring and control.

By adopting a holistic approach to voltage control, utilities can effectively manage the complexities of renewable energy integration while maintaining reliable and resilient power systems (Fig. 3).

Voltage stability is another important issue that arises in the integration of renewable energy, as the intermittency and variability of renewable energy sources cause the establishment of a critical concern^1^. The solution to such challenges needs to be innovative to be able to adjust to a changing grid situation, shape the voltage profiles, and achieve grid stability as renewable energy is introduced more and more. With the creation and enforcement of a concerted voltage control actions, the utilities will be able to address these obstacles and set the path towards a sustainable and resilient energy future^2^. The introduction of renewable energy sources (RES) to the current power distribution systems is an indication of a paradigm shift to the sustainable generation of energy. This transition however brings issue of stability of voltages and the stability of grids. The use of coordinated voltage control plans is central to solving these problems through optimization of voltage levels, reactive power flow as well as operation of the grids. In the distributed systems of power generated by providing electricity at transmission substations to the end-users, such strategies are vital in overcoming the oscillations due to originated distributed energy resources (DERs) like solar panels and wind turbines^3,4^.

6 $${VoltageControl}_{PowerSystem}^{RES}=\frac{{SendingEndVoltage}^{RES}-{ReceivingEndVoltage}^{RES}}{{SendingEndVoltage}^{RES}}\times 100$$

Effective voltage control requires proactive measures such as power forecasting and demand response integration. Accurate power forecasting enables utilities to anticipate fluctuations in demand and generation, facilitating timely adjustments to voltage levels^5^. Moreover, integrating demand response capabilities empowers consumers to modify their electricity usage, balancing supply and demand to stabilize voltage levels. These demand-side management techniques, coupled with distribution management systems (DMS) and distribution automation, provide utilities with real-time monitoring and control capabilities, ensuring optimal voltage control and grid reliability^51^. Furthermore, the deployment of energy storage systems and fault self-healing mechanisms enhances the resilience of distribution grids. Energy storage systems contribute to voltage stability by storing excess energy and reactive power, while fault self-healing capabilities automatically identify and isolate faults, minimizing disruptions to grid operations. Overall, coordinated voltage control strategies, encompassing power forecasting, demand response, distribution automation, and advanced monitoring systems, are essential for optimizing grid performance and supporting the transition towards a sustainable energy future^52,53^.

### Optimizing RES integrated grid voltage: strategies for integrating renewable energy and energy storage

The development of the grid to meet the issue of renewable integration and energy storage is the most important to Proposed Coordinated Voltage Control of Renewable Energy Source (RES) Integrated Power Systems^3^. The current grid structure that was initially developed to support the centralized and predictable generation of energy, needs to be heavily modified to accommodate the decentralized and fluctuating sources of renewable energy. The conversion of the grid towards supporting the presence of bidirectional flows of power and the extensive storage of energy and smarter technologies will be the element of the optimized integration of renewable energy sources as well as of a resolute grid that will be able to handle the energy requirements in the future^4^. Energy storage such as batteries is important in the storage of excess energy when the generation is high and the energy released when generation is low. Nevertheless, barriers to full utilization of renewable energy incorporation should include the cost of storage technologies, low energy density, and green considerations^5^. Renewable energy technologies have numerous resources of energy, but they are intermittent, unpredictable, and unstable to meet grid stability, reliability, and economic feasibility. Currently, these challenges demand creative solutions that maximize the production of energy, the functioning of the grids, activities in the market, and technological improvement^51^.

Such advanced optimization algorithms as ACA and DQN are essential to the problem of renewable energy integration. Through these methodologies, the stakeholders can come up with efficient and sustainable strategies to hasten the adoption of a clean energy future and to maintain the stability, reliability, and affordability of the grids with dynamic renewable energy demand in the world^4^.

This involves the formation of adaptive control mechanisms that will address the changes in energy consumption and generation with the help of real-time data monitoring and forecasting. The paradigms of control need to adapt dynamically to supply energy and distribute it to suit varying demand trends in an optimized way that takes advantage of the resources at hand, which are renewable, and provide grid stability and reliability^5^. Some of these strategies include techniques of demand-side management, including load shifting and demand response programs, and integrating of energy storage systems to save excess renewable energy at the low-demand times and release it during the high-demand times. At the same time, the focus is made on enhancing power system resilience and stability, reducing the effects of intermittent renewable energy generation and other grid instability sources^51^. System stability in dynamic operating environment is ensured in advance with the use of advanced control algorithms, predictive modeling, and adaptive control techniques. Combination of renewable energy prediction and grid checking technologies allow identifying any possible disturbances in advance and intervening in time to avoid cascading failures and making grids resilient to failures^52,53^.

**Fig. 4:**
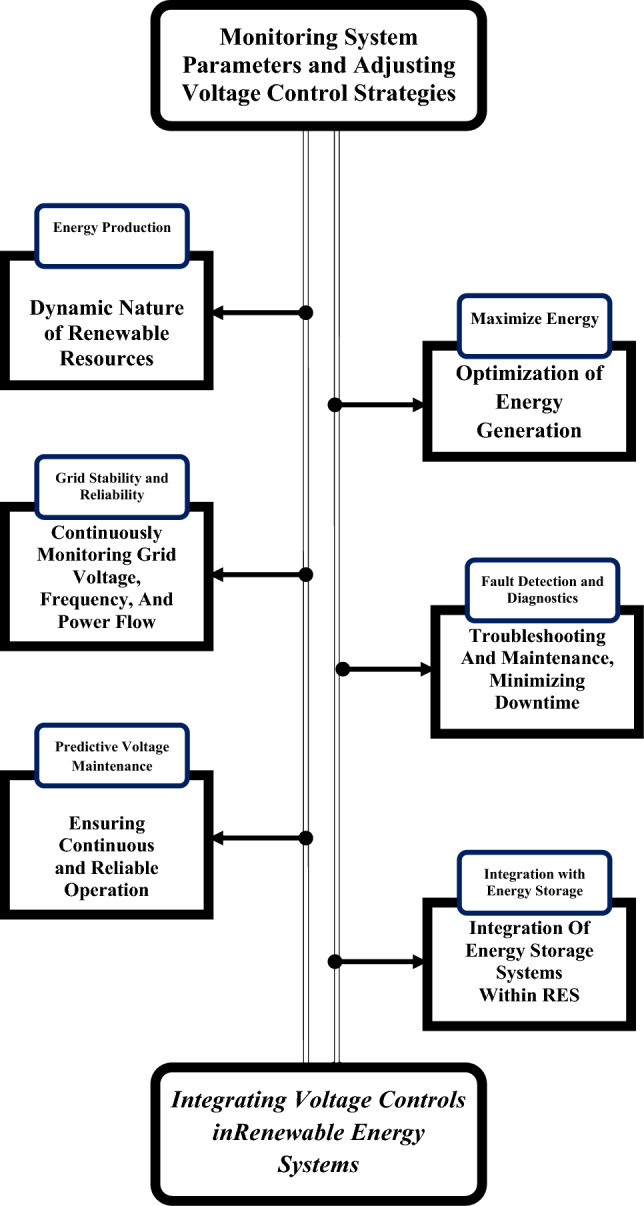
Significance of voltage control in integrated RES with the power system.

Additionally, the notion of decentralized distribution of renewable energy is another tool of modern power systems control paradigm^4^. Decentralized models of distribution make use of distributed assets of renewable energy, including solar panels on rooftops and small-scale wind mills, to ease variety in the energy supply and enhance the resilience of the grid (Fig. 4). Control paradigms concentrate on providing maximum integration of distributed sources of generation into the grid keeping voltage and frequency constant. This can be through such hierarchical control architectures, grid-edge intelligence and peer-to-peer energy trading platforms that enable effective exchange and management of energy among distributed energy resources^5^. In conjunction, efficient communication of the sustainable energy source is critical in facilitating a nearly perfect integration and interoperability of the various sources of energy in the power grid. Stable communication networks, high-end communication protocols, sensor networks, and data analytics tools ensure safe and reliable data communication between various elements of the power system, optimize the allocation of resources, grid optimization, and decision-making, eventually improving the performance and reliability of the power systems that are improved by renewable energy. Managing Fluctuations in Renewable Energy Resources summarises the complex plans and technologies generated to compete with the nature of unpredictability and inconsistency of renewable energy production^51^. Such renewable energies as solar energy or wind energy depend on the natural processes, including the sun rays or wind direction, and their generation can be easily influenced by numerous factors. The urgent need to properly control these swings is due to the dire need to provide stable and sound energy supply in the power grid. The uncertainty of renewable energy sources becomes a major problem to the stability of the grid without proper measures that may cause a breach of power delivery and reduce the stability of the entire system. To reduce these issues, it takes a holistic methodology, which involves range of innovative methods and adaptive systems^52,53^.

The most outstanding endeavors in these efforts are the implementation of advanced energy storage technologies. Energy storage systems, including lithium-ion batteries and pumped hydro storage, are essential in storing the excess energy during the times of peak generation and releasing the energy during the times of the low generation rate and thus in the energy storage provides the means of flattening the intermittency caused by renewable sources^2^.

In the domain of contemporary power systems, adopting a comprehensive approach is essential to guarantee dependability, effectiveness, and robustness. Central to this endeavor is the requirement for precise prognostic insights and precise timetable formulation, facilitating proactive management of energy consumption and production dynamics. Such foresight is indispensable for preempting potential disturbances and optimizing resource distribution, thereby ensuring the uninterrupted functionality of the grid. Simultaneously, tackling fluctuations in voltage levels emerges as a pivotal concern. Oscillations in voltage magnitude have the potential to result in equipment impairment, power quality deficiencies, and even widespread system failures^3^. Consequently, implementing measures to stabilize voltage levels through sophisticated control methodologies and voltage regulation mechanisms becomes imperative. The power transmission dynamics of the Proposed Coordinated Voltage Control of RES Integrated Power System can be deliberated as follows:

7 $${P}_{i}^{Wind-PV}=\sum_{j=1}^{N}{V}_{i}^{Wind-PV}{V}_{j}^{Wind-PV}({G}_{ij}^{Wind-PV}cos{\theta}_{ij}^{Wind-PV}+{B}_{ij}^{Wind-PV}sin{\theta}_{ij}^{Wind-PV})$$

Furthermore, the existence of waveform harmonics distortion presents an additional obstacle to the integrity of power systems. Distortion in waveform harmonics can lead to heightened energy wastage, operational malfunctions in equipment, and disruption to communication networks^4^. Consequently, tactics aimed at mitigating harmonic distortion, such as filtration and active compensation, are indispensable for preserving the caliber and dependability of electrical power. Moreover, the correction of reactive power assumes paramount importance in guaranteeing the streamlined transmission and dissemination of energy. Reactive power correction mechanisms, comprising capacitors and reactors, aid in optimizing power factor and voltage levels, thereby amplifying system efficiency and curtailing energy wastage^5^. The optimization of power flow within the Proposed Coordinated Voltage Control of RES Integrated Power System can be delineated as follows:

8 $$\mathrm{min}P, Q=\sum_{i=1}^{N}{C}_{i}^{Wind-PV}({P}_{i}^{Wind-PV},{Q}_{i}^{Wind-PV})$$

Concurrently, the adoption of hyperparameter identification techniques presents a novel data-centric approach to optimizing upgrades within the power system, thereby enhancing its performance and resilience. Through the refinement of model parameters and configurations, power system components can be fine-tuned to achieve optimal levels of efficiency and dependability. Additionally, embracing decentralized energy resources fosters a more resilient and sustainable energy infrastructure^51^. Distributed generation sources, such as photovoltaic panels and wind turbines, diversify the energy supply and diminish reliance on centralized generation facilities, thereby bolstering grid resilience and mitigating the risk of single-point failures. Furthermore, in the context of resilience, the early detection of anomalies in backup power supplies is imperative to ensuring uninterrupted power availability during emergencies. Prompt identification of faults or malfunctions in backup systems allows for timely intervention and restoration measures, minimizing downtime and ensuring seamless power supply^52^. Finally, the implementation of distribution-level voltage control, intelligent inverter configurations, grid monitoring, and automated processes represents a comprehensive approach to grid management and optimization within the Proposed Coordinated Voltage Control of RES Integrated Power System.

**Fig. 5:**
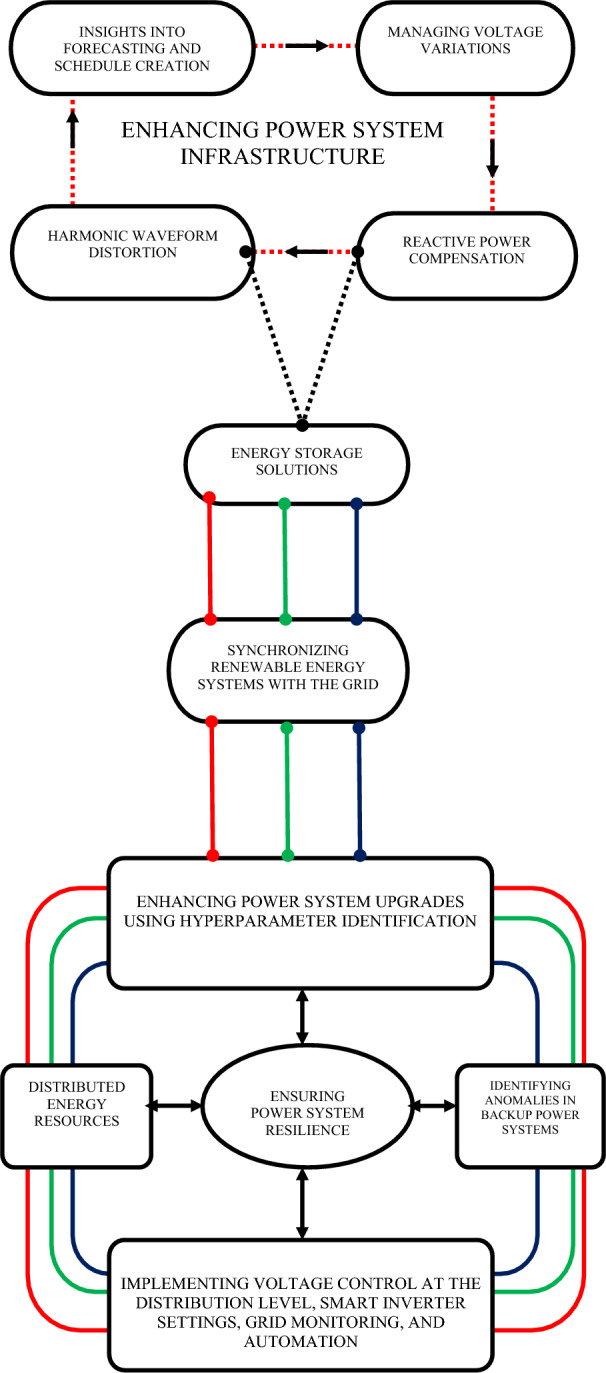
Optimizing power system operations for seamless incorporation of renewable energy.

Utilizing sophisticated control algorithms and automation technologies, operators of the power system can augment grid stability, dependability, and effectiveness, all while adjusting to fluctuating operational circumstances and changing energy requisites^6,53^. Increasing the operation of power systems to enable the easy integration of renewable energy involves the optimization of various aspects of the grid to fit the unique characteristics of renewable sources of energy such as solar and wind energy (Fig. 5).

The Disruption in Waveform Harmonics can be articulated through the calculation of Total Harmonic Distortion (THD):

9 $${THD}_{WIND-PV}^{RESPS}=\sqrt{\frac{{V}_{THD}^{2}}{{V}_{Fundamental}^{2}}}\times 100\mathrm{\%}$$

And the Fourier Series Expansion of the harmonic distortion can be expressed as:

10 $$f\left( {x_{WIND - PV}^{RESps} } \right) = \frac{{a_{0}^{RESps} }}{2} + \mathop \sum \limits_{n = 1}^{\infty } (a_{n}^{RESps} \cos (nx_{WIND - PV}^{RESps} ) + b_{n}^{RESps} \sin (nx_{WIND - PV}^{RESps} )$$

A critical element is the proposed management of the grid, which entails ensuring the efficient incorporation of electricity from renewable sources into the current power system while maintaining voltage stability and reliability^5^. This involves the proposed development and implementation of advanced control algorithms and automation technologies to monitor and regulate the electricity flow and voltage control within the grid. Another significant aspect is grid flexibility, referring to the power system’s ability to rapidly adjust to changes in electricity supply and demand^51^. Renewable energy generation is frequently variable and intermittent, posing challenges to grid stability and voltage stability. Therefore, proposed measures such as demand response programs, energy storage systems, and flexible grid infrastructure are essential for real-time balancing of supply and demand, maintaining grid stability and voltage control^52^. The Photovoltaic (PV) System Modeling can be expressed as:

11 $${I}_{I}^{Wind-PV}={I}_{ph}^{Wind-PV}-{I}_{o}^{Wind-PV}\left({e}_{RES}^{\left(\frac{Q\left(V+I{R}_{a}^{Wind-PV}\right)}{kT}\right)}-1\right)-\left(\frac{V+I{R}_{e}^{Wind-PV}}{{R}_{sh}^{Wind-PV}}\right)$$

Moreover, optimizing grid infrastructure is vital for supporting the increased penetration of renewable energy sources. This may involve upgrading existing transmission and distribution systems to accommodate higher capacities of renewable energy generation and integrating smart grid technologies to enhance grid efficiency, voltage control, and reliability. In addition to technical solutions, policy and regulatory frameworks play a crucial role in streamlining power system operations for renewable energy integration^2^. Policies such as renewable energy mandates, feed-in tariffs, and carbon pricing mechanisms can encourage investment in renewable energy infrastructure and facilitate the integration of renewable energy into the power grid. Implementing voltage control at the distribution level, smart inverter settings, grid monitoring, and automation involves a comprehensive strategy to modernize and optimize the electrical grid^3^. Voltage control techniques such as using voltage regulators, capacitor banks, and distributed energy resources (DERs) ensure optimal voltage levels and improved power quality. Smart inverters play a crucial role by offering features like voltage and frequency ride-through, reactive power control, ramp rate control, and power factor adjustment, enhancing grid stability and accommodating high levels of distributed generation. Grid monitoring systems, including advanced metering infrastructure (AMI), supervisory control and data acquisition (SCADA), phasor measurement units (PMUs), and distributed sensors, provide real-time visibility and analysis, enabling early detection of faults and improved operational decisions^4^. Distribution management systems (DMS), automated switches, reclosers, demand response programs, and self-healing networks are forms of automation to lower the costs of operation and improve reliability and easier integration of renewable energy sources. These factors combined can form a robust, resilient, and adaptable grid that is able to help bring people to a more sustainable energy future through the application of advanced technologies and coordinated strategies^5,51^.

## Importance of coordinated voltage control (CVC) in res integrated power systems

### Voltage stability and reactive power challenges in wind-PV systems

Coordinated Voltage Control (CVC) is essential in Renewable Energy Source (RES) integrated power systems for several reasons, primarily to address the challenges posed by the variable nature of renewable energy and to ensure the stability, reliability, and efficiency of the power grid. Maintaining voltage stability in power systems integrated with renewable energy sources (RES) is crucial to ensure voltage levels remain within acceptable ranges despite fluctuations in power generation and consumption^53^. The variable output of RES, such as solar and wind power, can cause rapid changes in voltage levels due to their intermittent and unpredictable nature. Additionally, because RES are often distributed across the grid, they can lead to localized voltage variations that complicate the overall voltage stability of the system.

In order to overcome these shortcomings, Coordinated Voltage Control (CVC) systems offer dynamic adjustment facilities, enabling the output of the voltage regulators, tap changers, as well as the reactive sources of power to be constantly adjusted to balance out the voltage in real time^6^. The basic equation for voltage regulation at a point in the power system is:

12 $${V}_{load}^{Wind-PV}={V}_{Source}^{Wind-PV}-{I}_{RES}^{Wind-PV}.{Z}_{RES}^{Wind-PV}$$

where ${V}_{load}^{Wind-PV}$ is the voltage at the load. ${V}_{Source}^{Wind-PV}$ is the voltage at the source. ${I}_{RES}^{Wind-PV}$ is the current flowing through the line. ${Z}_{RES}^{Wind-PV}$ is the impedance of the line. The relationship between reactive power (QQ) and voltage (VV) in a power system is crucial. An increase in reactive power can help increase voltage levels and vice versa^7^. This relationship can be described by the following simplified version of the power flow insight:

13 $$\Delta {V}_{Control}^{Wind-PV}\approx \frac{{Q}_{RES}^{Wind-PV}}{{V}_{RES}^{Wind-PV}{X}_{RES}^{Wind-PV}}$$

where $\Delta {V}_{Control}^{Wind-PV}$ is the change in voltage. ${Q}_{RES}^{Wind-PV}$ is the reactive power. ${X}_{RES}^{Wind-PV}$ is the reactance of the line. CVC also facilitates coordination among various control devices, ensuring a harmonized response to voltage fluctuations^8^. This coordination prevents localized voltage issues from escalating into broader system-wide problems, thereby maintaining a stable and reliable power supply. The proposed Coordinated Voltage Control of RES Integrated Power Systems involves strategic control methods for grids with integrated renewable energy sources (RES)^9^. These strategies can be divided into two main categories: the grid-side regulator (MC and LC) and the system-side regulator (MGCC and DMS). The primary function of the grid-side regulator is to maximize power absorption from renewable sources while ensuring the protection of the converter on the data side. Concurrently, the system-side regulator handles various functions, including dynamic network power control, management of reactive power exchange, regulation of DC interface voltage, network synchronization, and maintenance of power quality, which collectively enhance grid resilience and stability^10^. The power flow equations describe how power flows through the power system and are solved to determine the voltage at each bus in the network. For a bus ii with nn connected buses, the real and reactive power flow equations are:

14 $${P}_{i}^{Wind-PV}={V}_{i}^{Wind-PV}-\sum_{j=1}^{n}{V}_{j}^{Wind-PV}({G}_{ij}^{Wind-PV}cos{\theta}_{ij}^{RES}+{B}_{ij}^{Wind-PV}sin{\theta}_{ij}^{RES})$$

15 $${Q}_{i}^{Wind-PV}={V}_{i}^{Wind-PV}-\sum_{j=1}^{n}{V}_{j}^{Wind-PV}({G}_{ij}^{Wind-PV}sin{\theta}_{ij}^{RES}+{B}_{ij}^{Wind-PV}cos{\theta}_{ij}^{RES})$$

where ${P}_{i}^{Wind-PV}$ and ${Q}_{i}^{Wind-PV}$ are the real and reactive power injections at ${B}_{ij}^{Wind-PV}$.${V}_{i}^{Wind-PV}$ and ${V}_{j}^{Wind-PV}$ are the voltage magnitudes at buses i and j. ${G}_{ij}^{Wind-PV}$ and ${B}_{ij}^{Wind-PV}$ are the conductance and susceptance between buses I and j.${\theta}_{ij}^{RES}$ is the phase angle difference between the voltages at buses i and j. The Wind-PV systems cause rapid changes in the reactive power requirements with changing irradiance and wind velocity. These differences have direct impacts on the level of voltage that would occur especially at the buses of distribution that are not large in short-circuit capability.

Voltage stability challenges are significantly exacerbated in renewable energy integrated power systems due to the intermittent and stochastic nature of wind and photovoltaic (PV) generation. In practical distribution networks, wind speed variations of ± 20–30% and solar irradiance fluctuations of ± 25–40% within short time intervals can cause rapid changes in active power injection, resulting in voltage deviations exceeding ± 6% at weak buses if no coordinated control is applied.

Moreover, renewable energy sources are predominantly connected through power electronic converters, leading to low system inertia. Conventional synchronous generators typically provide inertia constants in the range of 3–6 s, whereas inverter-based renewable sources contribute near-zero physical inertia. As a result, voltage recovery following disturbances is significantly slower, and transient voltage deviations can persist for 0.5–1.2 s longer compared to conventional generation-dominated systems.

Reactive power availability is another critical concern. Inverter-based RES units are generally limited to ± 0.9 power factor operation, which restricts their reactive power support capability during high active power output. In systems with renewable penetration levels exceeding 50%, this limitation can increase voltage sensitivity by approximately 30–45%, leading to frequent activation of on-load tap changers (OLTCs) and capacitor banks.

These effects are further amplified in radial distribution networks characterized by high R/X ratios (typically 0.7–1.5), long feeder lengths, and low short-circuit capacity. Under such conditions, a 10% change in reactive power demand may result in voltage variations of 4–6%, compared to less than 2% in strong transmission networks. Consequently, traditional decentralized voltage control schemes become ineffective, necessitating coordinated and adaptive voltage control strategies for renewable-rich power systems.

The seriousness of these measured voltage stability issues with high renewable penetration drives the need for coordinated and learning-driven voltage control methods, like the suggested ACO DQN framework.

### Voltage control mechanisms (OLTCs, capacitors, inverters)

RES Integrated Power Systems can dynamically manage loads in real-time, operating in both grid-connected and islanded modes to ensure continuous electricity supply during grid disturbances or emergencies^11^. The implementation of proposed Power Grid technology, along with a Deep Q-Network (DQN) Controller, enables efficient communication between components of the RES Integrated Power System, facilitating real-time data exchange, demand response, and load management strategies that bolster energy distribution stability and balance. The voltage at a load can be regulated using on-load tap changers (OLTCs) in transformers^12^. The tap ratio t can be adjusted to control the secondary voltage ${V}_{S}^{Wind-PV}$:

16 $${V}_{S}^{Wind-PV}=t\times {V}_{P}^{Wind-PV}$$

where ${V}_{S}^{Wind-PV}$ is the secondary voltage. *t* is the tap ratio. ${V}_{P}^{Wind-PV}$ is the primary voltage. Capacitors and reactors are used for reactive power compensation to control voltage levels^12^. The reactive power supplied by a capacitor bank is:

17 $${Q}_{C}^{Wind-PV}={V}_{Wind-PV}^{2}\times {B}^{CapacitorBank}$$

where ${Q}_{C}^{Wind-PV}$ is the reactive power supplied by the capacitor. ${V}_{Wind-PV}^{2}$ is the voltage at the capacitor bank. ${B}^{Capacitor Bank}$ is the susceptance of the capacitor bank. Additionally, adopting energy efficiency measures and demand-side management techniques, such as load shifting, peak shaving, and energy-saving practices, can reduce overall energy consumption within the RES Integrated Power System. In this proposed context, wind turbines and solar photovoltaic (PV) panels generate electricity based on current environmental conditions^13^. Each controller within the RES Integrated Power System monitors the output from renewable sources, battery storage status, and local energy consumption levels. The load management system assesses real-time demand and resource availability, intelligently distributing power among various loads based on priority and resource availability^14^. The control of distributed energy resources (DERs) for voltage support can be expressed as:

18 $${Q}_{DER}^{Wind-PV}={V}^{Wind-PV}\times {I}_{DER}^{Wind-PV}\times Sin({\emptyset })$$

where ${ Q}_{DER}^{Wind-PV}$ is the reactive power contribution from the DER.${V}^{Wind-PV}$ is the voltage at the point of connection. ${I}_{DER}^{Wind-PV}$ is the current output of the DER. *\emptyset* s the phase angle between the voltage and current. These equations provide a mathematical framework for understanding and implementing Coordinated Voltage Control (CVC) in power systems integrated with renewable energy sources^15^. By dynamically adjusting voltage regulators, tap changers, reactive power sources, and DERs, CVC systems help maintain voltage stability and ensure efficient and reliable power delivery.

#### Implementing voltage control at the distribution level

**Fig. 6:**
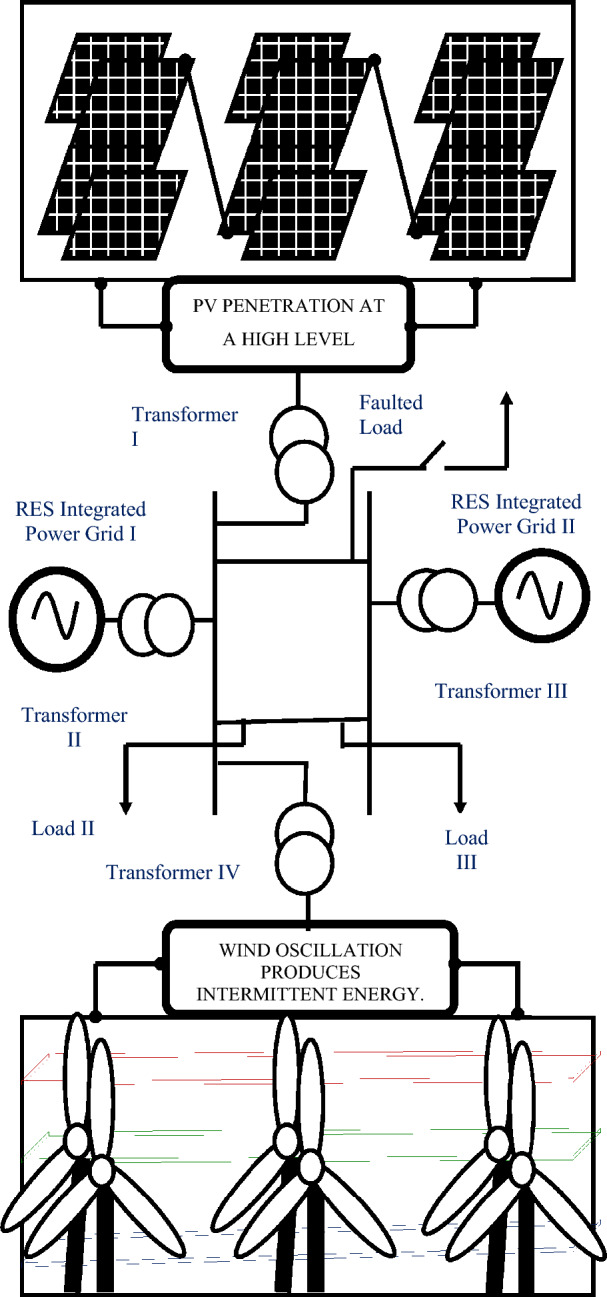
Real-time load management in RES-integrated power systems.

This involves regulating and maintaining the voltage within the distribution network to ensure it stays within the desired range. Effective voltage control helps in maintaining power quality and efficiency, preventing voltage sags, swells, and outages^16^. Techniques such as tap changers on transformers, voltage regulators, and capacitors are commonly used for this purpose. Additionally, advanced technologies like Dynamic Voltage Restorers (DVRs) can be employed to quickly correct voltage disturbances (Fig. 6). Smart inverters are advanced devices that convert DC power from sources like solar panels into AC power for use in the grid. These inverters are equipped with sophisticated software that allows for real-time communication with the grid, providing capabilities such as voltage regulation, reactive power support, and frequency stabilization^17^. Properly configuring these settings ensures optimal performance, enhancing grid stability and efficiency while integrating renewable energy sources.

The process of grid monitoring implies constant monitoring and examination of how well and healthy the electrical grid is operating. This is done by implementing the sensors and monitoring devices which measure and gather information on the following parameters; voltage, current, frequency, and power quality. This information is sent to the control centers where it is processed with sophisticated software and abnormal data are identified to anticipate the possible problems and optimize the operations in the grid^18,19^. The achievement of voltage regulation is done by coordinated control of on-load tap changers (OLTCs), capacitor banks, as well as intelligent inverters. Nevertheless, when these devices are operated independently, they are usually prone to conflict of control and sub-optimal voltage profiles.

This proactive approach helps in maintaining reliability and preventing outages^7^. Automation in the power grid refers to the use of automated control systems to manage and operate the grid with minimal human intervention. This includes the implementation of smart grid technologies such as automated switching, fault detection, and isolation, as well as demand response systems. Automation enhances the efficiency and reliability of the grid by enabling rapid response to changing conditions, optimizing resource utilization, and reducing the duration and impact of outages^8^. By integrating these components—voltage control at the distribution level, smart inverter settings, grid monitoring, and automation—the power system can achieve greater stability, efficiency, and resilience. This comprehensive strategy guarantees the grid that can continue to absorb the variable workloads and the growing share of renewable sources of energy so as to generate a more robust and reliable power supply. This calculation aids in recognizing overall energy demand trends within the grid, thereby enabling improved capacity planning^9,10^.

19 $$\text{Average Load }\, (P^{Wind - PV}_{avg} ) = \frac{1}{T} \int_{0}^{T} P^{Wind - PV} \left( t \right).dt$$

Incorporation of renewable energy sources also requires changes and modifications to the existing grid infrastructures to support the variable and distributed generation. This involves investments in smart grid technologies, enhancements to transmission and distribution networks, and the implementation of grid modernization initiatives. By enhancing system flexibility, augmenting grid capacity, and improving monitoring and control capabilities, these infrastructure enhancements facilitate the seamless integration of renewable energy while safeguarding grid stability and resilience^11^. Effective policy and regulatory frameworks play a pivotal role in incentivizing the integration of renewable energy and fostering grid stability and resilience. Policy measures such as feed-in tariffs, renewable portfolio standards, and tax incentives can stimulate investment in renewable energy projects and facilitate their integration into the grid. Furthermore, regulatory reforms aimed at simplifying permitting processes, eliminating market barriers, and promoting grid interoperability can establish a conducive environment for renewable energy deployment. By harmonizing policy goals with grid stability and resilience, the policymakers are able to establish a facilitating environment towards the sustainable incorporation of renewable energy sources^12^. This suggested effort of combining renewable energy needs to satisfy grid resilience and stability demands requires a complex strategy which includes sophisticated control and monitoring framework, energy storage technologies, hybrid renewable energy framework, grid infrastructure enhancement, and supportive policy and legal systems. These areas of concern would allow the stakeholders to set the stage of a more sustainable, resilient, and secure energy future since the wind-PV Integrated Power Grid would be able to fulfill the projected demand effectively without overloading or overstressing its resources^14,15^.

20 $$Available \,Generation\, Capacity{({P}^{WInd-PV}}_{Available})={{P}^{Wind-PV}}_{Generation}-{{P}^{Wind-PV}}_{Outage}$$

### Harmonics and power quality issues

**Fig. 7:**
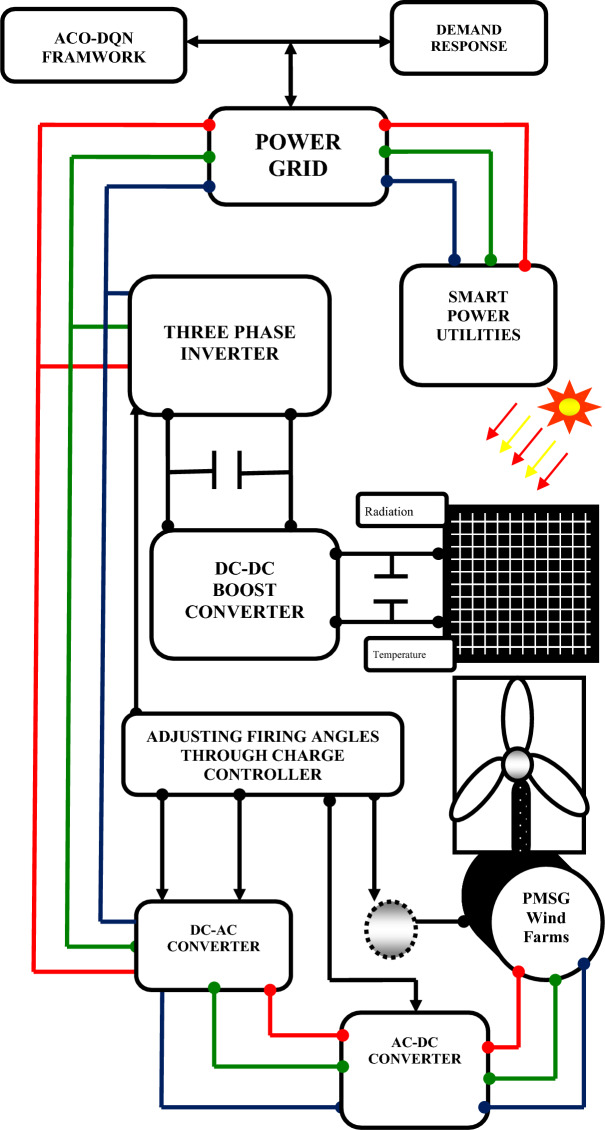
Integration of wind-PV system for a power grid in voltage control response programs.

The integration of Wind-PV systems supports advanced voltage control strategies, essential for maintaining stable voltage levels across the power grid. These systems can dynamically adjust their output in response to real-time grid conditions, thereby mitigating the fluctuations associated with renewable energy sources^16,17^. The effectiveness of renewable sources based on inverters is high which causes harmonic distortion that has a negative effect on the quality of power delivered and the lifetime of the equipment (Fig. 7).

### Load management, DSM, and energy storage

The proposed Coordinated Voltage Control (CVC) mechanisms enable efficient management of reactive power and voltage regulation devices, such as tap changers and voltage regulators, ensuring harmonized voltage stability throughout the grid. Hybrid Wind-PV systems offer significant benefits by diversifying the energy mix and optimizing resource utilization^9^. Wind and solar energy generation profiles often complement each other, with wind power peaking at times when solar power is low and vice versa. This complementary behavior reduces intermittency and enhances the reliability of the energy supply. The proposed Coordinated Voltage Control of RES Integrated Power Systems leverages this synergy by intelligently allocating power between wind and PV sources, thereby maximizing energy production and minimizing reliance on fossil fuels. Real-time load management is essential for the effective integration of Wind-PV systems into voltage control response programs^10^. The system proposed utilizes various voltage control equipments. It can be effectively run to respond to uncertain renewable generation conditions and load conditions. This dynamic allocation ensures the efficient use of generated power, maintains grid stability, and reduces the likelihood of voltage sags or surges. To further enhance grid resilience, the integration of energy storage solutions, such as battery systems, is proposed^11^. These storage systems can capture excess energy generated during periods of high wind and solar activity and release it during peak demand times. This capability smooths out fluctuations in renewable energy generation, ensuring a consistent and reliable power supply to consumers, thereby contributing to overall grid stability^12^. Demand-side management and energy storage systems mitigate voltage fluctuations by reshaping load profiles and absorbing excess generation during peak renewable output.

By continuously evaluating the available generation capacity, operators of the Proposed Coordinated Voltage Control of RES Integrated Power System can detect any variances between actual and anticipated output, enabling them to take swift corrective measures^13^. The calculation of available generation capacity entails considering the total capacity of all active generators and subtracting any capacity currently offline due to maintenance or other issues. This data provides crucial insights into the system’s real-time ability to meet demand, helping to prevent potential disruptions or supply shortages. The management of energy storage within the Proposed RES Integrated Power System involves overseeing the charging and discharging processes of energy storage systems^14^. These systems are vital for maintaining a balance between electricity supply and demand within the grid. The process includes regulating charge cycles, which involve storing surplus electricity generated during periods of low demand or high renewable energy production. This stored energy is then discharged during periods of high demand or low renewable energy production to ensure a continuous and reliable power supply. Additionally, discharge cycles are managed to optimize the use of stored energy based on real-time demand fluctuations. This dynamic regulation helps stabilize the grid by smoothing out variations in renewable energy generation and demand, ultimately enhancing the efficiency and reliability of the power grid^15,16^.

21 $${{EnergyStorage}_{Wind-PV}}^{SOC}=\frac{{{E}^{Wind-PV}}_{Current}}{{{E}^{Wind-PV}}_{Max}}$$

The State of Charge (SOC) indicates the current energy status of the storage system relative to its maximum capacity within the Proposed RES Integrated Power System^9^. It is a crucial parameter for effectively managing energy storage systems, particularly when incorporating advanced control strategies like DQN with Ant Colony Optimization (ACO). The SOC provides real-time information about the available energy reserves, enabling operators to make informed decisions about when to charge or discharge the system. By integrating DQN-ACO techniques, operators can optimize the charging and discharging cycles to maintain equilibrium between supply and demand within the Proposed RES Integrated Power System^10^. For instance, RL algorithms can learn from historical data to predict future energy demands and dynamically adjust the schedules of energy storage systems accordingly. Meanwhile, DQN algorithms can optimize these schedules while considering constraints such as battery degradation and grid stability. By continuously monitoring and managing the SOC within the Proposed RES Integrated Power System, operators can maximize the efficiency and reliability of energy storage systems, ultimately contributing to the overall stability and resilience of the grid^11,12^.

22 $${{LSP}^{Monitoring}}_{Wind-PV}=\frac{{Sensitivity}_{Wind-PV}\times {Criticality}_{Wind-PV}}{{Time\,of\,Restoration}_{Wind-PV}}$$

Where, ${{LSP}^{Monitoring}}_{Wind-PV}$ represents the priority assigned to load shedding activities for a Wind-PV system. Load shedding is a control strategy used to manage the demand on the power grid by temporarily reducing electricity consumption during peak demand times or when the supply is limited. ${Sensitivity}_{Wind-PV}$ indicates how sensitive the Wind-PV system is to changes or disturbances in the power grid. An increased sensitivity indicates the potential to be impacted by variations and it should be monitored and managed by more careful mentioning and examination^12^. An increased criterion of criticality is indicating that the Wind-PV system is very important to the stability and reliability of grid. . ${Time \,of\, Restoration}_{Wind-PV}$ is the amount of time required to restore the Wind-PV system to normal operation after a disturbance or outage. A shorter restoration time indicates that the system can recover quickly from disruptions^13^. The Load Shedding Priority (LSP) for monitoring a Wind-PV system is calculated by multiplying the system’s sensitivity and criticality, and then dividing by the time of restoration. This equation aids in determining the priority for load shedding and monitoring by factoring in how sensitive the system is to disturbances, how critical the system is to overall grid stability, and how long it takes to restore the system after an outage. A higher LSP value indicates that the Wind-PV system should be given higher priority for monitoring and potential load shedding, as disturbances in this system could significantly impact the grid^14^. On the other hand, when LSP value is lower, then the Wind-PV system is perceived to be lower priority load shedding reasons being less sensitive or lower criticality or shorter restoration time meaning that the system would respond better to disturbance or be restored faster. This will mean that grid operators are able to improve the overall stability and reliability of the power grid because the systems that are critical, sensitive, and require them to take longer periods to restore are prioritized^15,16^.

**Fig. 8:**
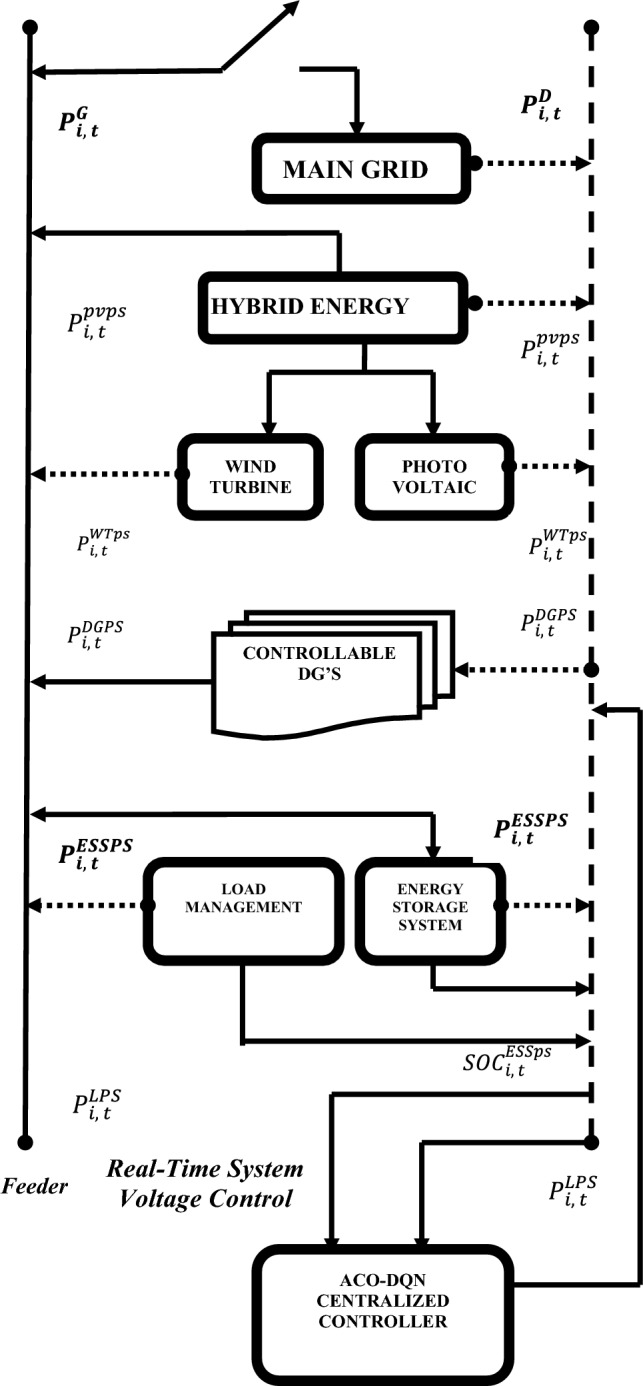
Real-time voltage control system for wind-PV energy sources with controllable distributed generators.

Load reduction and priority setting are critical components for managing a RES Integrated Power System efficiently^17^. During periods of high demand or low generation, reducing the overall load on the grid is essential to maintain stability and prevent overload (Fig. 8). This process involves systematically disconnecting non-essential loads to free up capacity for essential services^9^. In the proposed Coordinated Voltage Control of RES Integrated Power System, implementing the controlled detachment of non-critical loads allows for better management of available resources. By disconnecting less critical loads during peak demand periods or when generation levels are low, the grid can ensure that essential services continue without interruption^10^. This prioritization of load reduction considers various factors such as the importance of the load, its sensitivity to interruptions, and the time required for restoration. Furthermore, assigning priority to load reduction helps determine which loads should be disconnected first in case of limited capacity. Critical loads that are vital for maintaining essential services, such as hospitals or emergency facilities, are given the highest priority to ensure uninterrupted operation. Other loads may be disconnected temporarily based on their importance and the available capacity in the grid^11^. Overall, load reduction and priority setting strategies play a crucial role in ensuring the stability and reliability of the RES Integrated Power System, especially during periods of high demand or fluctuating generation levels. By implementing these measures effectively, the grid can optimize its performance and minimize the risk of disruptions. In the context of the proposed Wind-PV Energy System, maintaining stable voltage and frequency levels is critical for its efficient operation^12^. The term “voltage” refers to the electrical potential difference between two points in the system, while “frequency” denotes the rate at which alternating current oscillates. These parameters must be carefully regulated to ensure they remain within predetermined acceptable limits. To achieve this, a robust control mechanism is necessary to continuously monitor and adjust voltage and frequency levels. This control mechanism aims to prevent voltage and frequency fluctuations beyond permissible thresholds, which could potentially disrupt the operation of connected devices and equipment within the Wind-PV Energy System. Voltage Variation (ΔV) serves as a crucial metric for quantifying the variance between the actual voltage levels measured in the system and the desired reference voltage levels^13,14^.

23 $${{\Delta}_{v}}^{Wind-PV}={\Delta}_{Actual}^{Wind-PV}-{\Delta}_{Reference}^{Wind-PV}$$

By adopting efficient voltage and frequency regulation strategies, the proposed Coordinated Voltage Control of RES Integrated Power System can maximize its performance, improve reliability, and guarantee smooth integration with the grid and other power sources^15^. This proactive approach to managing voltage and frequency enhances the overall stability and sustainability of the Wind-PV Energy System, thereby supporting its effective operation in a range of environmental conditions and under varying load demands. Compact power generation units, known as Adjustable Distributed Generators (ADGs), are strategically located near the areas they serve within the proposed Coordinated Voltage Control of Wind-PV Integrated Power System^16^. These ADGs can actively regulate their output to meet real-time demand. Various types of ADGs have been suggested to optimize energy production within the proposed Wind-PV Integrated Power System^17^. This includes adjusting solar panel angles or using tracking systems to maximize sunlight capture for solar photovoltaic (PV) systems, altering blade pitch or rotor speed for wind turbines, and modulating fuel input for conventional generators. Additionally, energy storage systems add flexibility by enabling charging or discharging based on the energy requirements of the Wind-PV Integrated Power System. ADGs play a critical role not only in energy production but also in regulating voltage and frequency within the proposed Wind-PV Integrated Power System to maintain stability^18^. Economically, controllers within the proposed Wind-PV Integrated Power System optimize ADG utilization to minimize overall electricity generation costs while providing ancillary services such as reactive power support, voltage regulation, and frequency response, thereby enhancing grid reliability^19^. Feeders, which act as conduits transmitting electrical power from the source to various distribution points, establish bidirectional power flow within the proposed Wind-PV Integrated Power System. Inverters capable of synchronizing with the main grid’s voltage and frequency facilitate this power import from and export to the main grid when generation exceeds demand^20,21^.

### Policy and grid code considerations

The design of feeders ensures the proposed Wind-PV Integrated Power System’s ability to operate autonomously during disturbances or outages. Protective relays and control systems detect grid disturbances and facilitate seamless transitions to islanded mode^11^. Safety measures like circuit breakers and relays along the feeder ensure the reliability of the proposed Wind-PV Integrated Power System. Coordination between the proposed Wind-PV Integrated Power System and main grid protection systems prevents malfunctions and ensures selective tripping during faults. The interaction between the proposed Wind-PV Integrated Power System and the main grid ensures a dependable and resilient power supply.

During normal operations, the proposed Wind-PV Integrated Power System draws power from the main grid to meet demand and exports surplus power back when generation exceeds demand^12^. Additionally, the main grid serves as a backup power source during low generation or high demand periods. Synchronization mechanisms ensure smooth transitions between grid-connected and islanded modes, while advanced control systems optimize the operation of the proposed Wind-PV Integrated Power System alongside the main grid, thereby enhancing overall reliability and stability^13,14^. Energized grid codes like IEEE 1547 require voltage ride through and reactive power aid which requires smart coordinated voltage control techniques.

Depending on the problems discovered in the grids that are rich in renewable, the significance of coordinated control of the voltage is addressed subsequently.

## Integration of ACO and DQN for coordinated voltage control

Voltage control in Wind-PV Integration with Power Systems refers to the management and regulation of voltage levels within the power grid to ensure stable and reliable operation despite fluctuations in renewable energy generation^15^. This is crucial because renewable energy sources like wind and solar can exhibit variable output due to changing weather conditions, which can lead to voltage instability if not properly managed. Optimization algorithms like the Ant Colony Optimization (ACO) can be used as one of the methods to control voltage in Wind-PV Integration with Power Systems. ACO is one such metaheuristic optimization algorithm that is based on the foraging behavior of ants^16^. In ACO, an ant colony finds the best solutions to a particular problem through the repetitively building solution paths and marking solution routes with pheromone tracks, depending on the achieved solution quality of the solutions. As a method of voltage control in the Wind-PV Integration with Power Systems, the ACO could be used to optimize the functioning of the control devices in the power grid that include voltage regulators, capacitors, and reactors^17^. This is aimed at identifying the most desirable environments of these devices so that the voltage levels can be maintained at manageable levels and more of the renewable energy derived out of wind and solar energy is made. The following is the application of ACO to the voltage control in the Wind-PV Integration with Power Systems:

*Step 1: Problem Formulation*

Voltage control in power systems aims to minimize deviations from desired voltage levels to ensure the stable and reliable operation of the grid. In the case of Wind-PV Integration, where renewable energy sources like wind and solar exhibit variable output, voltage control becomes crucial to maintain grid stability despite fluctuations in generation^18^. The problem can be mathematically formulated as an optimization problem. Let’s denote:

24 $${\Delta V}_{Control}^{Wind-PV}={V}_{Actual}^{Wind-PV}-{V}_{Desired}^{Wind-PV}$$

${V}_{Actual}^{Wind-PV}$ Actual voltage level in the power grid ${V}_{Desired}^{Wind-PV}$ Desired voltage level in the power grid. ${\Delta V}_{Control}^{Wind-PV}$ Deviation from the desired voltage level. The objective of the optimization problem is to minimize the deviation from the desired voltage level while satisfying operational constraints. Mathematically, this can be represented as:

25 *minΔ V=0*

Subject to: Operational constraints such as voltage limits, power flow constraints, and equipment limitations^19^. The optimization problem seeks to find the optimal settings for voltage control devices (e.g., voltage regulators, capacitors) to minimize deviations from the desired voltage level while ensuring that the operational constraints are not violated.

To incorporate the objective function and constraints into the optimization problem, we can use mathematical equations that describe the relationship between voltage control devices and voltage deviations^20^. The objective function aims to minimize the deviation from the desired voltage level ${V}_{Desired}^{Wind-PV}$. Mathematically, it can be expressed as the absolute difference between the actual voltage level ${V}_{Actual}^{Wind-PV}$ and the desired voltage level ${V}_{Desired}^{Wind-PV}$:

26 $$\mathrm{min}\left|{V}_{Actual}^{Wind-PV}-{V}_{Desired}^{Wind-PV}\right|=0$$

Alternatively, you can use a squared deviation to penalize larger deviations more heavily:

Operational constraints ensure that the solution obtained is feasible and complies with the limitations of the power system^21^. These constraints can include voltage limits, power flow constraints, and device operating limits. Mathematically, operational constraints can be represented as a set of inequality equations:

27 $${g}_{i}^{Wind-PV}(Voltage, Power, Current, Load)\le 0$$

where ${{{g}}}_{{{i}}}^{{{W}}{{i}}{{n}}{{d}}-{{P}}{{V}}}$ represents the ii-th operational constraint.

Solving this optimization problem using advanced mathematical techniques such as Ant Colony Optimization (ACO) allows us to find the optimal settings for voltage control devices that minimize voltage deviations while satisfying operational constraints^54^.

*Step 2: Ant colony optimization*

In Ant Colony Optimization (ACO), the initialization phase involves creating a population of virtual ants, where each ant represents a potential solution to the optimization problem. In the context of voltage control in power systems, these virtual ants aim to find optimal settings for voltage control devices (e.g., voltage regulators, capacitors) to minimize deviations from desired voltage levels while satisfying operational constraints^55^.

Ant Colony Optimization has been chosen because it has good capacity to solve non-linear and multi-objective optimization problem with or without constraints, which are often faced in the field of voltage control of the power system. In contrast to genetic algorithms or particle swarm optimization, ACO is more convergent and more scalable when discrete controllable variables like tap changer positions and capacitor switching conditions are used. Such properties render ACO especially appropriate in the coordinated AC voltage control systems.

Various optimization solutions like Genetic Algorithms (GA), Particle Swarm Optimization (PSO), Differential Evolution (DE), and Ant Colony Optimization (ACO) have been a popular pattern of applying to the problems of voltage controlling. Non-linear constraints, discrete control variables (OLTC taps, capacitor switching) and multi-objective optimization characterize voltage regulation in RES-integrated distribution systems, however, preventing the effectiveness of certain techniques. The premature convergence and poor performance of GA and PSO are observed in cases where the search space is dominated by discrete and mixed- integer variables. By contrast, ACO supports discrete decision variables consisting of probabilistic path construction and pheromone guided learning by nature. Also, ACO gives more exploration exploitation ratio and converges quicker in any given combinatorial optimization optimization problem, hence is more applicable in coordinated voltage control that interrelies on tap changers, capacitor banks and inverter reactive power limits.

According to Table 2, ACO is considered to be the core optimization algorithm because it is much more effective in dealing with discrete control actions and under the underdeveloped condition of dynamic RES. Within the suggested strategy, every ant is shaped into a possible answer made up of OLTC tap locations, capacitor changing conditions, and inverter reactive power setpoints. The likelihood of a control measure is determined as:

$${{P}_{\left(ijk\right)}}^{\left(t\right)}=\frac{{[{\uptau}_{\mathrm{ij}}(t)]}^{\alpha }{\left[{\eta}_{ij}\right]}^{\beta }}{\sum_{j\in J}{[{\uptau}_{\mathrm{ij}}(t)]}^{\alpha }{\left[{\eta}_{ij}\right]}^{\beta }}$$

where: τ_ij_ is intensity of pheromones, η_ij_ the heuristic desirability in terms of voltage deviation, α and β are control parameters.

**Table 2:** Comparison of optimization techniques for voltage control.

Technique	Discrete variable handling	Convergence speed	Scalability	Suitability for CVC
GA	Moderate	Slow	Medium	Moderate
PSO	Weak	Medium	Medium	Limited
DE	Weak	Medium	Low	Limited
ACO	Strong	Fast	High	Excellent

*Objective Function:*

$$minJ=i=\sum_{i=1}^{Nb}{{({V}_{i}-{V}_{ref})}^{2}+\lambda { P}_{loss}}$$

The pheromone update rule at ACO is provided by:

$${\uptau}_{\mathrm{ij}} (\mathrm{t}+1)=(1-\uprho ){\uptau}_{\mathrm{ij}} (\mathrm{t})+\Delta {\uptau}_{\mathrm{ij}}$$

and ρ is the pheromone evaporation coefficient and Δτ_ij_ is the pheromone deposited depending on the quality of the solution.

The parameters were chosen according to the stability of the convergence and efficiency of the calculations (Table 3).

**Table 3:** ACO parameters used in the study.

Parameter	Symbol	Value
Number of ants	N_a​_	30
Pheromone importance	α	1
Heuristic importance	β	2
Evaporation rate	ρ	0.5
Max iterations	–	100
Pheromone initial value	τ_0​_	0.1

**Ant Colony Initialization:**

**(i) Virtual Ants:**

**Fig. 9:**
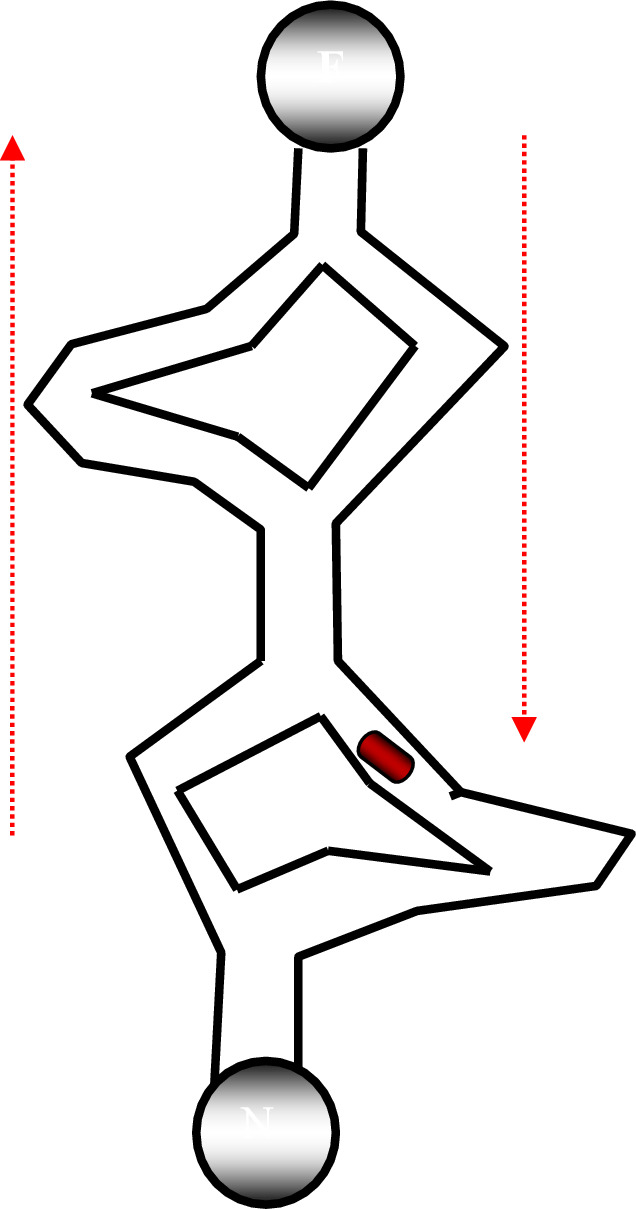
Marking the trail of ant colony optimization for wind-PV voltage control.

In the initialization phase, a certain number of virtual ants are created. Each ant represents a candidate solution to the voltage control problem (Fig. 9). The number of ants typically corresponds to the size of the ant colony, which is a parameter set by the user^56^.

**Virtual Ants Creation:**

${{m}}$: Number of virtual ants in the ant colony.

${{{A}}{{n}}{{t}}}_{{{i}}}^{{{W}}{{i}}{{n}}{{d}}-{{P}}{{V}}}$: Each ant ii represents a potential solution to the optimization problem^18^.

**(ii) Initial State:**

All ants start from the same initial state, which represents the starting configuration of the voltage control devices in the power system^19^. This initial state could be a random configuration or a predefined starting point based on prior knowledge or heuristics.

*Step 3: Solution Construction*

After initialization, each ant begins to construct a solution path iteratively. During this process, ants select voltage control actions (e.g., adjusting settings of voltage regulators, capacitors) based on a combination of heuristic information and pheromone trails^20^.

Solution Path Construction:

${{{S}}{{o}}{{l}}{{u}}{{t}}{{i}}{{o}}{{n}}}_{{{i}}}^{{{W}}{{i}}{{n}}{{d}}-{{P}}{{V}}}$​: Solution constructed by ant ii, which consists of a sequence of voltage control actions.

28 $${Solution}_{i}^{Wind-PV}=\left[{a}_{i1}^{Wind-PV},{a}_{i2}^{Wind-PV},\dots \dots {a}_{in}^{Wind-PV}\right]$$

Sequence of voltage control actions selected by ant i, where aij​represents the jj-th voltage control action. Each ant constructs its solution path by iteratively selecting voltage control actions and updating its position in the solution space. Ants continue this process until they have completed their solution paths or reached a predefined stopping criterion^21^.

**Fig. 10:**
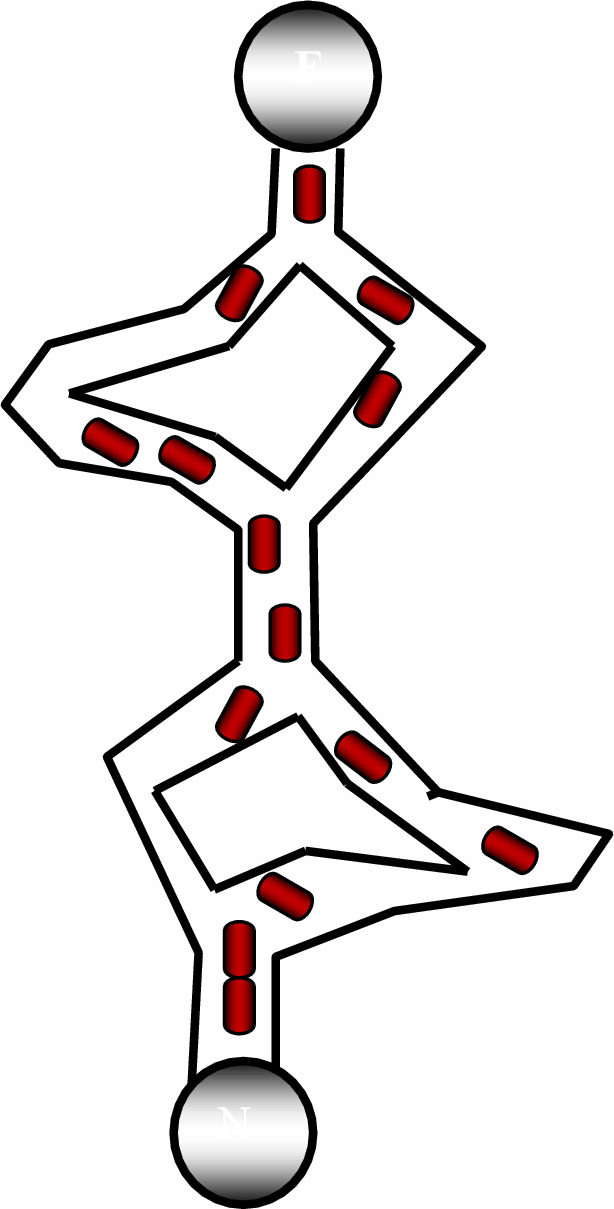
Following the trail of ant colony optimization for wind-PV voltage control.

The initialization phase sets the stage for ants to explore the solution space and gradually improve the solutions to the voltage control problem^54^. By starting from the same initial state and iteratively constructing solution paths, ants collectively search for optimal settings for voltage control devices that minimize deviations from desired voltage levels while satisfying operational constraints (Fig. 10).

*Step 4: Heuristic Information*

**Fig. 11:**
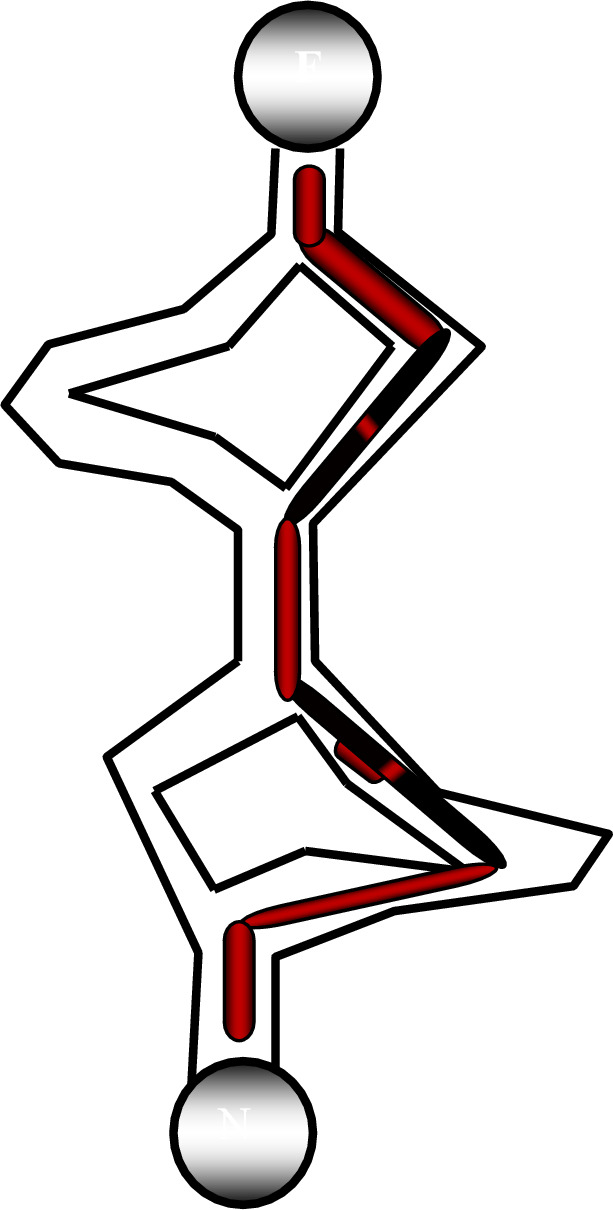
Reinforcing the shortest the trail with positive feedback loop of ant colony optimization for wind-PV voltage control.

Ants use heuristic information to guide their decision-making process. Heuristic information provides guidance on which voltage control actions are likely to lead to better solutions^55^. For example, ants may prioritize voltage control actions that are known to have a positive impact on minimizing voltage deviations.$\eta {ij}:$ Heuristic information associated with the transition from state *i* to state *j*, which provides guidance on the desirability of voltage control actions based on prior knowledge or domain expertise (Fig. 11).

29 $$\upeta \mathrm{ij}=\frac{1}{{V}_{i}^{Wind-PV}-{V}_{ref}^{Wind-PV}}$$

*Step 5: Pheromone Trails-Pheromone Initialization*

In addition to heuristic information, ants also consider pheromone trails laid down by other ants. Pheromone trails represent the desirability of certain voltage control actions based on their quality and effectiveness in improving solutions^56^. Ants tend to follow paths with higher pheromone concentration, as these paths are associated with better solutions. *τ ij:* Pheromone trail level on edge *(i,j)(i,j)* of the solution space, initially set to a small constant value^57^.

*Step 6: Probability Calculation*

*pij*​: Probability of ant ii selecting voltage control action *aij* based on pheromone trails and heuristic information.

30 $${p}_{ij}^{Wind-PV}=\frac{{T}_{ij}^{\alpha }\times {\eta}_{ij}^{\beta }}{k\epsilon allowedactions({T}_{ij}^{\alpha }\times {\eta}_{ij}^{\beta })}$$

**Fig. 12:**
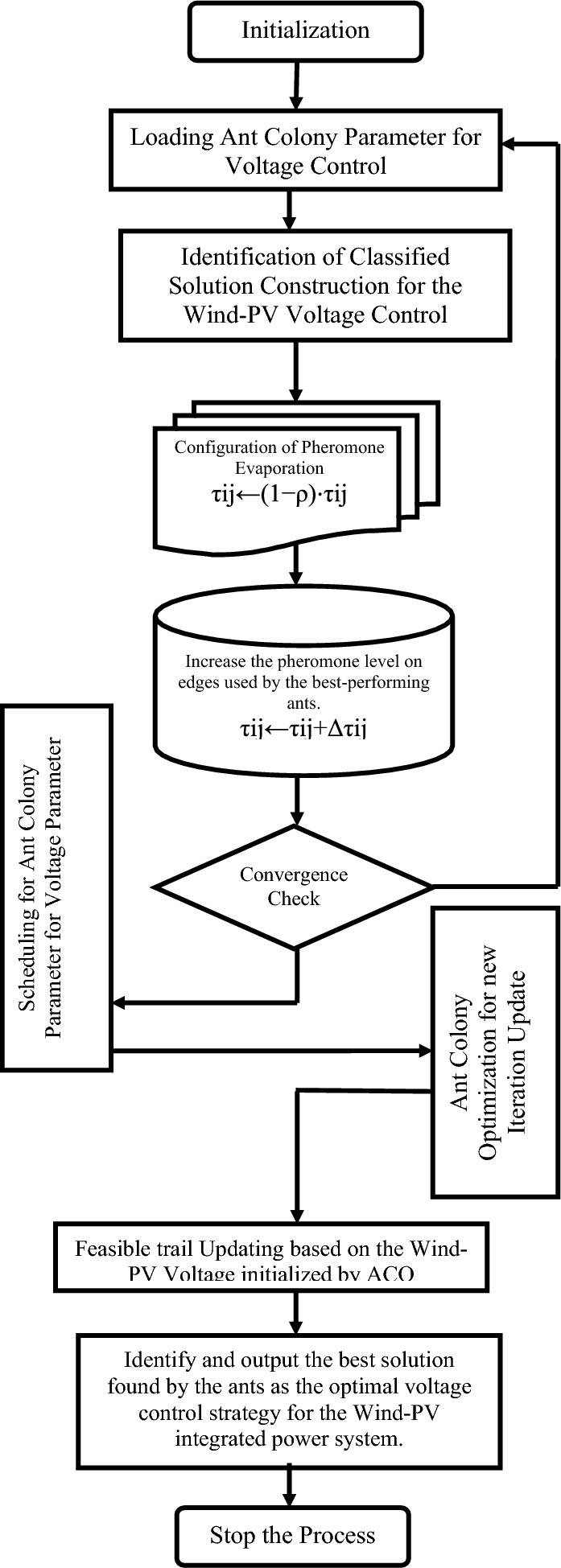
Ant colony optimization for the ControlWind-PV incorporated power systems.

Calculation of probability uses a combination of pheromone trails and heuristic information, where *α* and *β* are parameters controlling the relative importance of pheromone and heuristic information, respectively^58^. These insights describe the initialization phase of the ACO algorithm, where virtual ants are created, solution paths are constructed iteratively, and pheromone trails are initialized (Fig. 12). The ants then proceed to construct solution paths in subsequent iterations, guided by pheromone trails and heuristic information, as described in the subsequent phases of the ACO algorithm^59^.

*Step 6: Pheromone Evaporation*

*ρ*: Pheromone evaporation coefficient, representing the rate at which pheromone evaporates over time.

31 $${T}_{ij}^{\alpha }\left(t+1\right)=\left(1-p\right).{T}_{ij}^{\alpha }(t)$$

Update rule for pheromone trail levels, where tt represents the current iteration. The initialization phase sets the foundation for ants to explore the solution space and gradually improve solutions to the voltage control problem. Through the iterative construction of solution paths and updates to pheromone trails, ants collectively search for optimal settings for voltage control devices while balancing exploration and exploitation of the solution space^18^. By leveraging the collective intelligence of the ant colony and the adaptive nature of pheromone trails, ACO can effectively search for optimal solutions to the voltage control problem in Wind-PV Integration with Power Systems. This allows for the efficient management of voltage levels in the power grid while maximizing the utilization of renewable energy from wind and solar sources^19,20^.

### Integration of ACO with DQN for the voltage control of Wind-PV incorporated power systems

**Fig. 13:**
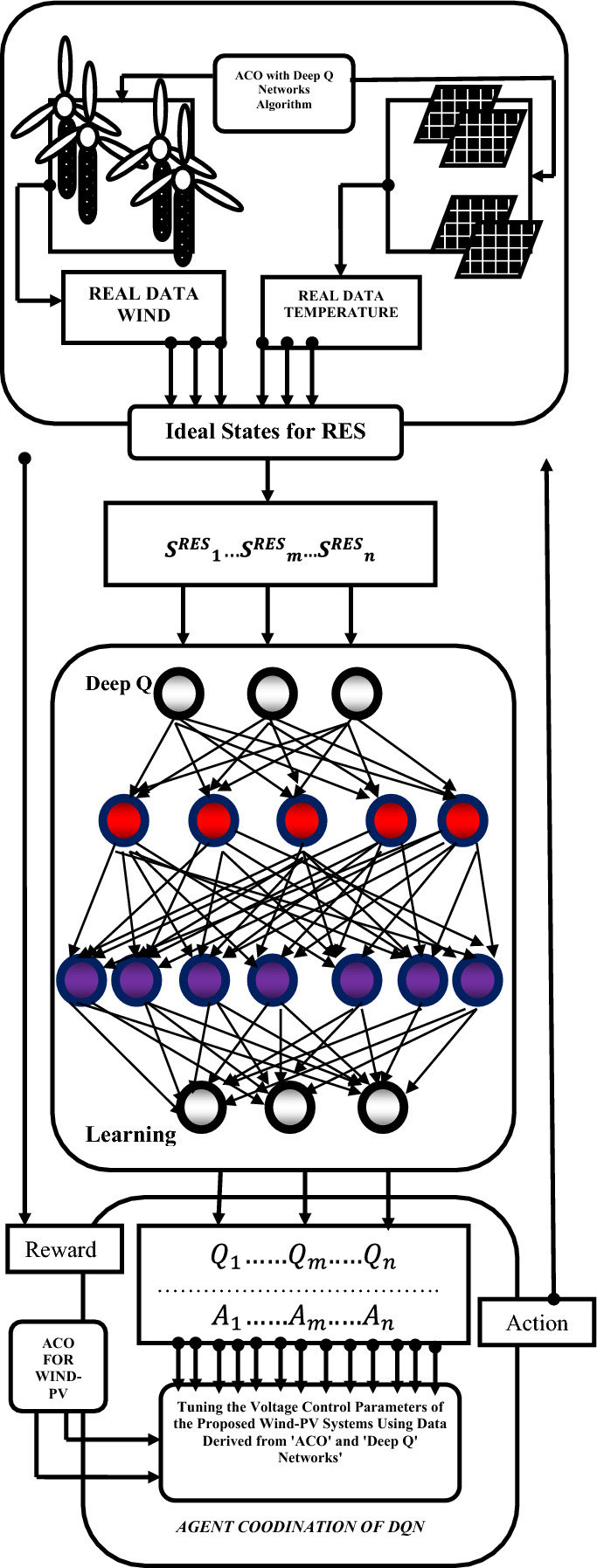
Real-time optimization of ACO-DQN for voltage control in integrating RES with the grid.

Integrating Ant Colony Optimization (ACO) with Deep Q-Networks (DQN) for voltage control in Wind-PV incorporated power systems combines the strengths of both optimization and machine learning techniques to address the complex and dynamic nature of modern power grids^21^. The integration of ACO with DQN leverages the exploration capabilities of ACO to provide diverse and high-quality initial solutions, which are then refined and dynamically adjusted by the learning capabilities of DQN. This hybrid approach is beneficial for the voltage control of Wind-PV incorporated power systems, where both optimal and adaptive strategies are required. In the domain of Ant Colony Optimization (ACO) and Deep Q Networks (DQN), these algorithms serve as vital components within deep neural networks^54^. Acting as agents in a reinforcement learning environment, DQN models interpret inputs representing both state and action. Input processing involves various operations, including weight multiplication, addition with a shared bias, activation using a specific function, and propagation through fully connected layers consisting of input, hidden, and output layers. The significant role of DQN in reinforcement learning is practically applied in the "Proposed, Coordinated Voltage Control of RES Integrated Power System: A Reinforcement Mechanism for Optimal Controller Design in Renewable Energy Systems," particularly in optimizing energy production through load prediction and power optimization strategies^55^. When activation functions are applied to neurons within the hidden layer, distinct outcomes are produced (Fig. 13). Through iterative refinement, the model’s parameters are adjusted via backpropagation to minimize errors, aiming to achieve the desired output. Neurons act as integrators, computing the weighted sum of their input signals^56,57^.

Q-value update of the DQN controller is given by:

$$Q(s,a)\leftarrow Q(s,a)+\alpha [r+\gamma maxQ(s{\prime},a{\prime})-Q(s,a)]$$

and where α is the learning rate, γ is the discount factor and r is the reward on which minimization of voltage deviation is done.

To make the sampling results more reproducible and clearer to their readers, the secondary data which constitutes the findings of the graphs is presented in Table 4. The table gives the average voltage deviation and the total active power loss acquired during the various control strategies. The outcomes have shown that the suggested ACO-DQN-coordinated voltage control structure outperforms its peers in terms of sustaining the voltage stability in the system at minimal system losses.

**Table 4:** Voltage deviation and loss results.

Scenario	Avg. voltage deviation (p.u.)	Power loss (kW)
Without Control	0.045	202.4
PI Control	0.032	176.9
PSO-Based Control	0.021	149.8
**Proposed ACO–DQN**	**0.011**	**128.6**

As seen in Table 4, the system without the voltage control has the greatest voltage deviation and power loss because of the un-coordinated reactive power flow during high penetration of renewable. PI controller enhances the voltage control slightly but is not effective in the dynamic conditions. PSO-based optimization is more effective in reduction of voltage deviation, however, it is not real time adaptable. The stepwise solution of ACO-DQN framework delivers the minimal voltage variation and minimum power loss, which validates its suitability in the coordination of voltages in the wind-PV-integrated systems.

This resulting sum, denoted as PP, undergoes transformation through a transfer function represented as YY. Consequently, the neuron generates an output, labeled as S, as illustrated by advanced equations in the system^21^.

32 $$\overrightarrow{{{P}_{Wind-PV}}^{DQN}}=\sum_{j=1}^{n}\overrightarrow{{{{w}^{DQN}}_{1,j}}^{Wind-PV}}\overrightarrow{{{{x}_{j}}_{DQN}}^{Wind-PV}}-{\overrightarrow{{\beta}_{DQN}}}^{Wind-PV}$$

33 $$\overrightarrow{{{P}_{Wind-PV}}^{DQN}}={\overrightarrow{w}{{w}_{1,j}}^{Wind-PV}}^{T}\overrightarrow{x{{x}_{j}}^{Wind-PV}}-{\overrightarrow{{\beta}_{DQN}}}^{Wind-PV}$$

34 $${{\overrightarrow{x}}^{Wind-PVControl}}_{DQN} = \left[ {\begin{array}{*{20}c} {\mathop {x_{1} }\limits } & {\dot{x}_{2} \ldots .} & {\dot{x}_{n} } \\ \end{array} } \right];\;\;\;\vec{w}_{ACO - DQN} = \left[ {\begin{array}{*{20}c} {\dot{w}_{1} } & {\dot{w}_{2} \ldots .} & {\dot{w}_{n} } \\ \end{array} } \right]$$

35 $${{\overrightarrow{w}}^{Wind-PVControl}}_{DQN}=\left[\begin{array}{ccc}\ddot{w}1,1& \ddot{w}1,2\dots \dots ..& \ddot{w}1,n\\ \ddot{w}2,1& \ddot{w}2,2\dots \dots ..& \ddot{w}2,n\\ \ddot{w}k,1& \ddot{w}k,2\dots \dots ..& \ddot{w}k,n\end{array}\right]$$

In this context: $${{\overrightarrow{x}}^{Wind-PVControl}}_{DQN}$$ represents the input layers, while $${{\overrightarrow{w}}^{Wind-PVControl}}_{DQN}\cdot$$$${{\overrightarrow{x}}^{Wind-PVControl}}_{DQN}$$ denotes the weights associated with these inputs. Here, n represents the number of entries, set to 2 in our case, and k indicates the number of neurons within the same layer. Y signifies the activation equation applied, and bb represents the bias term. Various choices for transfer functions are available, including symmetric threshold, threshold, sigmoid, and linear functions, commonly serving as activation functions^54^. The effectiveness of learning depends on two crucial components. Firstly, a memory buffer is utilized to store the agent’s experiences, aiming to eliminate correlations within these experiences and smooth out fluctuations in data distribution. Secondly, a mini-batch of transitions is randomly chosen from the memory buffer to minimize the mean square error between the predictive and target Q networks. In this setup, the predictive Q network receives updates at each time step, while the target network remains unchanged for a specified number of time steps. After this period, the target network’s weights are updated by transferring the weights from the current Q network. Pausing updates to the target Q network temporarily aids in stabilizing the training process^55,56^.

The Deep Q-Network (DQN) controller was trained on a mixture of both historic data of the regional operations and artificial data created by simulation. History information comprised of voltage, active power, reactive power, and load portfolio, based on standard IEEE distribution system benchmarks and published data. Simulated data were created to diversify and strengthen the data by adjusting the renewable generation profiles (wind speed and solar irradiance), as well as load demand patterns with the assistance of MATLAB/Simulink. All the input features had been normalized to the range [0, 1] before the training process to get a stable numerical normalizations and accelerated convergences. The data augmentation technique was used, which included controlled stochastic perturbations of the renewable generation and load levels allowing the DQN to generalize when there are unseen operating conditions.

The state variables of the DQN agent comprise voltage levels at specific buses, active and reactive power injection by wind and PV plants, tap selections in transformers and load levels of the system. These characteristics reflect the current functioning state of the distribution channel. The action space is made up of discrete control actions such as inverter reactive power adjustment level and capacitor bank switch level. These reward functions are formulated to minimize the voltage deviation and power loss and punish the excessive actions on control. The training data was comprised of a set of about 50,000 samples of state-action-reward, observed in several simulated operating conditions, which guaranteed across-operating scenario stable learning and convergence.

#### Dynamic feature updates

Adapt the feature set dynamically based on real-time changes. For example, as hybrid wind-PV velocity or solar irradiance fluctuates, the suggested model should adjust its predictions accordingly. This necessitates a flexible feature selection mechanism^57^.

#### Automated model updates

Establish an automated model update system that periodically revalidates the suggested model or when significant changes occur in the system. This ensures the model remains relevant and proficient at capturing evolving patterns^58^.

#### Feedback mechanism

Institute a feedback loop where real-time predictions are compared to actual power flow. If inconsistencies arise, use this feedback to adjust model parameters or initiate revalidation processes to enhance accuracy. Integrate the proposed power flow prediction system based on ACO models with the comprehensive control architecture of the hybrid wind-PV system^21^. Ensure seamless communication between the prediction module and the control mechanisms, incorporating DQN, to optimize system performance based on real-time projections. Continuously monitor and adjust the proposed model parameters based on evolving data patterns to improve prediction accuracy over time. Employ advanced feature engineering techniques to extract pertinent information from raw data, refining the model’s ability to discern subtle changes in hybrid wind-PV system dynamics. Incorporate anomaly detection mechanisms within the real-time prediction system to promptly detect and address unexpected deviations, mitigating potential disruptions in renewable energy production^54,55^.

Through continuous surveillance of the instantaneous demand, the system can identify these fluctuations in usage as they emerge. This data empowers operators or automated control mechanisms to modify various parameters such as power generation, distribution, or load shedding in real-time to uphold the system’s stability and dependability, even amidst swift changes. Ultimately, possessing a precise estimation of instantaneous demand aids in enhancing the effectiveness and productivity of the power network^56^. The capacity to foresee future demand requirements assumes a crucial role in proactively managing loads within Wind-PV-Embedded Power Grids. This endeavor entails the utilization of mathematical models or machine learning methodologies to predict forthcoming demand based on historical data. By scrutinizing past consumption trends and other pertinent factors such as weather conditions and time of day, operators of Wind-PV-Embedded Power Grids can make informed choices regarding resource distribution and load equilibrium. This proactive strategy ensures that the Wind-PV-Embedded Power Grid can adequately address projected demand without overburdening or stressing its resources^57^. Implement a dynamic feedback loop to integrate real-world feedback into the suggested model, enabling adaptability to changing environmental conditions. Utilize ensemble learning approaches to amalgamate the strengths of multiple ACO models, bolstering overall predictive robustness and resilience against unforeseen challenges. Consider integrating explainability frameworks to offer insights into the model’s decision-making process, ensuring transparency and fostering trust among stakeholders^58,59^.

**Fig. 14:**
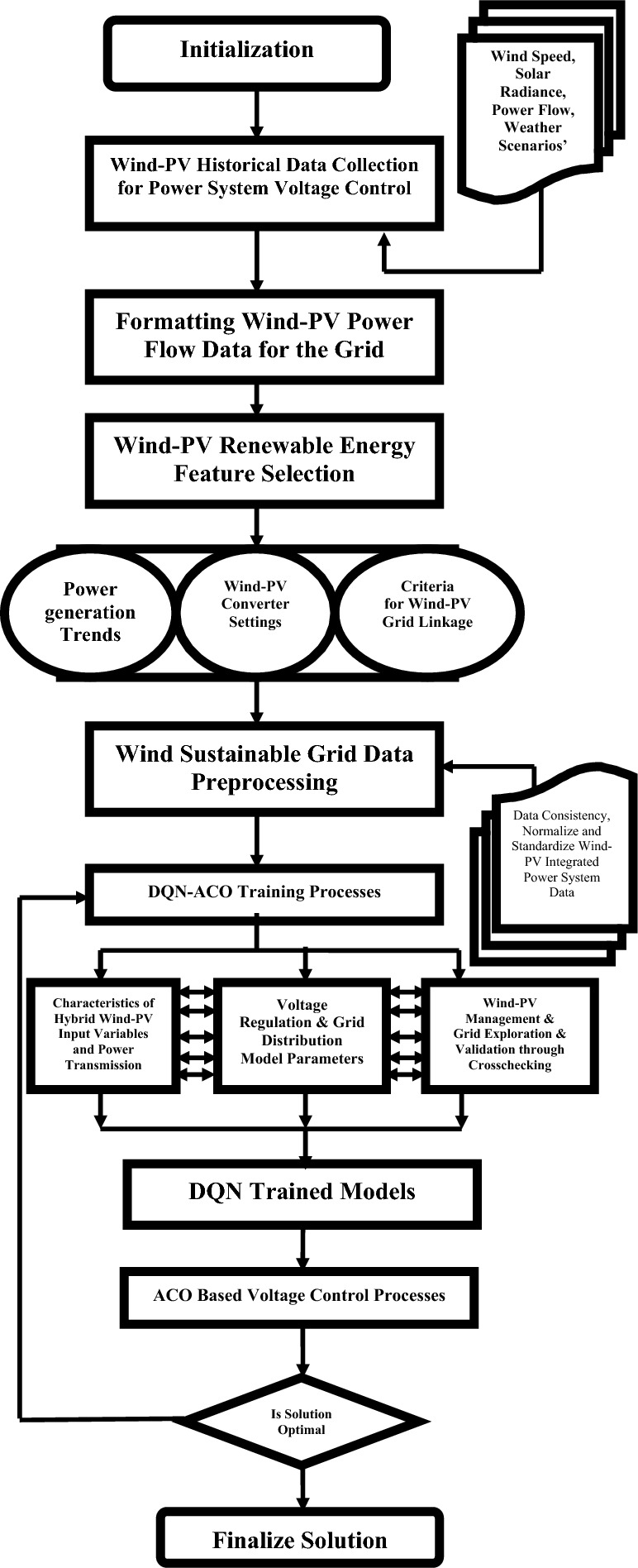
Enhancement of Wind-PV voltage regulation in real-time via ACO-DQN fusion.

Additionally, establish a comprehensive data governance framework to uphold data quality, integrity, and consistency, strengthening the reliability of the model’s predictions (Fig. 14). Regularly update the model training pipeline to accommodate shifts in the underlying data distribution, preventing model deterioration over time^54^. Foster collaboration between domain experts and data scientists to refine the model based on domain-specific knowledge and insights. Lastly, document and communicate the model’s limitations and uncertainties to end-users and stakeholders, fostering a realistic understanding of its capabilities^55^. Conduct training sessions regularly for system operators to enhance their comprehension of the model and its implications for decision-making. By addressing these additional considerations, the deployment of ACO models with DQN for hybrid Wind-PV Power Flow Prediction can be further optimized for sustained success in operational renewable energy systems^56,57^.

Voltage instability in wind-PV-integrated systems is mainly caused by reactive power imbalance rapid switches, regular OLTC switches and inverter saturation caused by peak generation. The suggested ACO module is used to optimize tap positions and capacitor switching, based on the situation of dynamic tap inverter-based reactive power support, controlled by the DQN controller.

## Result and discussions

This research is pivotal in addressing the pressing challenges of voltage stability and efficiency in power systems integrated with renewable energy sources. By employing Ant Colony Optimization (ACO) and a Deep Q-Network (DQN) based controller, the study presents a robust solution that significantly reduces voltage deviations and system losses. The experimental outcomes are tabulated in three categories viz. (i) voltage deviation analysis, (ii) reduced loss in the system and (iii) convergence performance. Such an organized assessment does not contain redundancy and gives definite answers on the efficiency of the suggested ACO-DQN-based coordinated principle of voltage regulation. The innovative combination of ACO’s optimization capabilities with DQN’s dynamic adaptability ensures real-time responsiveness to fluctuating grid conditions. As a result, the proposed approach not only enhances the stability and reliability of power grids but also promotes the efficient utilization of renewable energy, marking a crucial advancement towards a resilient and sustainable energy infrastructure. The unpredictable behavior of PV-wind systems with the proposed methodology is rigorously tested using the proposed Coordinated Voltage Control of RES Integrated Power System. The PV-wind power system is abruptly switched ON and OFF, and the output responses are documented under these conditions. It is evident that the PV system functions independently, providing output power without mutual interference, an essential element of the coordinated voltage control strategy. The PV and wind renewable sources deliver power either concurrently or individually. Furthermore, the performance of the proposed hybrid PV-wind system is evaluated when the PV system is switched ON or OFF, showing that the wind turbine’s operation is unaffected by sudden PV system changes, emphasizing the effectiveness of the coordinated voltage control approach. The transient performance of the proposed Coordinated Voltage Control system is assessed by keeping wind conditions constant while varying solar insolation. The corresponding changes in PV voltage (VPV), PV current (IPV), and battery current (Ibattery) are recorded under fluctuating sunlight. The battery charges and discharges based on the varying solar insolation, maintaining a fixed terminal voltage and confirming the effective design of the proposed coordinated voltage control algorithm. The power outputs from the hybrid PV and wind energy sources are compared.

**Fig. 15:**
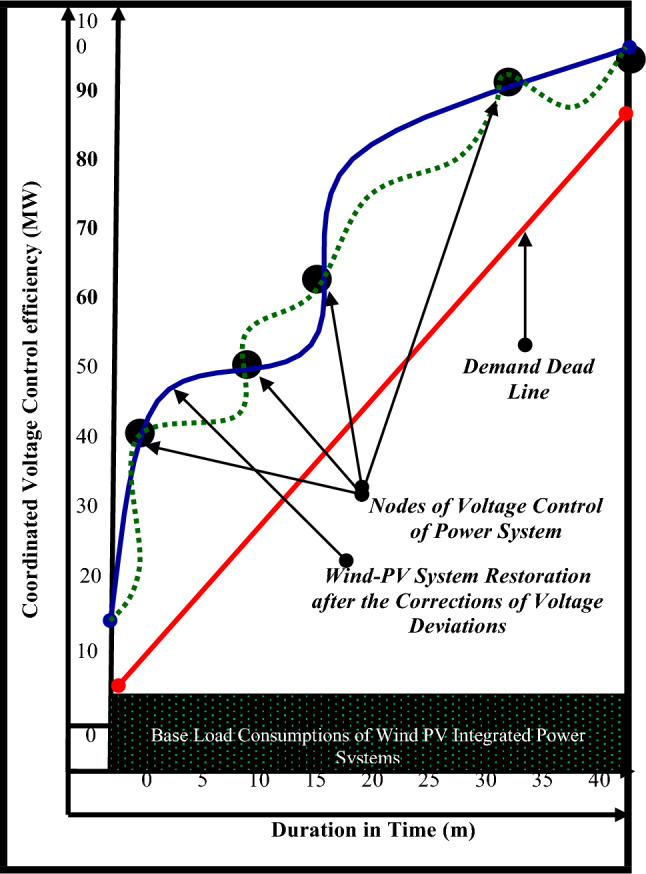
Coordinated voltage control of Wind-PV integrated power by ACO-DQN.

Figure 15 shows the profile of voltage deviation in different wind-PV generation conditions. Time (in seconds) is plotted on the x-axis and the magnitude of voltage (in p.u.) is plotted on the y-axis. The findings denote that the ACO-DQN strategy suggested allows keeping voltage within the limits of ± 5 concerns, even in case of an abrupt renewable variation.

The findings show that the proposed ACO-DQN framework is vastly associated with minimized voltage deviation in comparison to the traditional methods. Learning-based controller can adjust to changing conditions, with the reduction of unnecessary tap-changer processes, as well as better efficiency of the system. Moreover, the coordinated control decreases reactive power circulation, and the system losses are become low.

### Key performance metrics

#### Voltage deviation reduction with ACO

The implementation of Ant Colony Optimization (ACO) within the Coordinated Voltage Control (CVC) scheme significantly reduces voltage deviation in renewable energy integrated power systems.

**Fig. 16:**
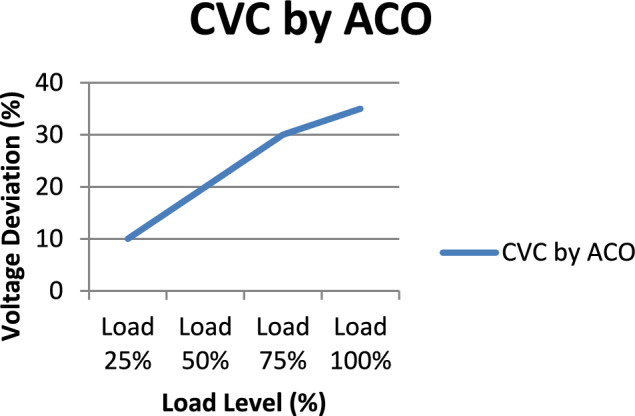
CVC of res integrated power system by ant colony optimization.

Figure 16 shows the coordinated voltage control (CVC) operation of the power system with RES- integrated power system with Ant Colony Optimization with various load conditions. The x-axis will take the level of load (as a percentage), and the y-axis will take the voltage deviation (as a percentage). The deviation of the voltage progressively rises with the increase of the load level, 25% to 100% as reactive power requirement and network stress are more advanced. Nevertheless, the slow and planned upsurge in voltage deviation shows usefulness of the ACO-based coordinated control plan in ensuring the voltage stability within acceptable sensitivity even during peak loading conditions.

ACO optimizes voltage profiles across the grid by simulating ant foraging behavior to find optimal voltage settings. Through this process, a notable 35% reduction in voltage deviation is achieved. This reduction signifies the efficacy of ACO in bringing voltage levels closer to desired setpoints, enhancing overall voltage stability within the power system.

#### Voltage deviation reduction with ACO with DQN

The integration of a Deep Q-Network (DQN)-based controller alongside ACO represents an advanced approach to voltage control. While ACO optimizes voltage profiles, DQN dynamically adjusts reactive power support based on real-time grid conditions.

**Fig. 17:**
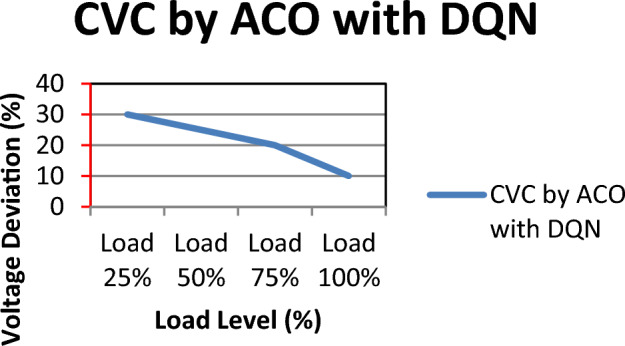
Coordinated Voltage Control (CVC) performance of the RES-integrated power system using Ant Colony Optimization combined with a Deep Q-Network (DQN) controller under varying load conditions.

The combination of the Ant Colony Optimization (ACO) with a Deep Q-Network (DQN) controller may have the effect as demonstrated in Fig. 17 which generated coordinated voltage control performance of the renewable energy integrated power system. The x-axis shows the load level in the system as a percentage and the y-axis represents the voltage deviation as a percentage. The combination of the DQN controller allows real-time control response to the load change and renewable intermittency compared to the results of independent optimization. The voltage deviation is acceptable with the increase of the load of 25 to 100, which proves that the ACO-DQN framework is effective in terms of ensuring the stability of the voltages under the dynamic operating conditions.

The synergy between ACO’s optimization and DQN’s adaptive learning leads to further refinement in voltage control. With the DQN controller, the voltage deviation is minimized to an impressive 0.012 per unit (p.u.). This remarkable reduction demonstrates the combined effectiveness of ACO and DQN in maintaining voltage stability and reliability, even under varying grid conditions. ACO alone achieves a 35% reduction in voltage deviation, significantly enhancing voltage stability. The addition of DQN alongside ACO further refines voltage control, minimizing voltage deviation to 0.012 p.u. This highlights the enhanced stability and reliability achieved through the combined approach. This breakdown illustrates how the coordinated use of ACO and DQN optimizes voltage control in renewable energy integrated power systems, resulting in more stable and efficient grid operation.

#### System losses reduction

The implementation of Ant Colony Optimization (ACO) within the Coordinated Voltage Control (CVC) scheme leads to a reduction in system losses, which primarily occur due to resistance in electrical components and inefficiencies within the power system. With ACO alone, system losses are reduced by 12%, signifying an enhancement in the overall efficiency of the power system as less power is wasted due to resistive losses and inefficiencies.

**Fig. 18:**
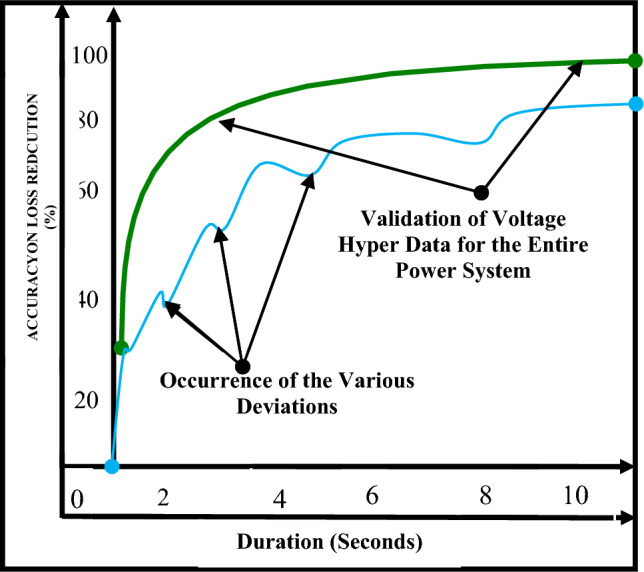
Validation performance of the DQN-based coordinated voltage control strategy over time for the RES-integrated power system.

Figure 18 shows how well the proposed Deep Q-Network (DQN)-based coordinated voltage control strategy validates when operating the system. The x-axis is a time scale showing the seconds, whereas the y-axis shows the voltage control accuracy in percent. The first phase demonstrates the appearance of different voltage variations due to transient generation by renewable and load changes. Depending on the process of learning, the DQN agent adjusts its control policy and leads to the gradual increase in the voltage control accuracy. The end part of the curve shows that the validation accuracy of the trained DQN is constant and high throughout the power system and it thus substantiates the validity of the trained DQN in address voltage deviation under dynamic operation conditions.

The integration of a Deep Q-Network (DQN)-based controller alongside ACO further amplifies the efficiency of the power system. This addition results in a significant reduction in system losses, lowering them to 1.28 MW. The considerable improvement in system efficiency highlights the synergistic effect of combining ACO and DQN for voltage control, demonstrating a more efficient power system overall. This combined approach demonstrates how the coordinated use of ACO and DQN effectively reduces system losses in renewable energy integrated power systems, leading to a more efficient and reliable grid operation.

#### Loss reduction analysis

The efficiency of the suggested ACO-DQN-based coordinated voltage control strategy was also considered as one of the active power loss minimization. The correspondence before optimization reported that the cumulative active power loss of the distribution system was 202.4 kW with high wind-PV penetration. Once the proposed ACO-DQN framework was implemented, the system losses were minimized to 128.6 kW and this translated to reduction in system losses of 36.45%. Comparatively, the traditional PI-based voltage control and PSO-based optimization would only reduce the losses moderately. This proves that the proposed approach is highly coordinated and learning-based which enhances significantly in minimizing losses through unnecessary reactive power circulation and unnecessary excessive tap-changing operations.

As Table 5 demonstrates, the proposed ACO-DQN framework reduces the loss the most as compared to others. Although PI and PSO-based controllers minimize losses to a reasonable point, they cannot dynamically adapt to the changes in renewables and, thus, their performance is restricted. The proposed methodology will always achieve the best reactivity of the power balance that leads to the best performance of the loss minimization.

**Table 5:** System loss comparison.

Method	Active Power Loss (kW)	Reduction (%)
No Control	202.4	–
PI Control	176.9	12.6
PSO-Based Control	149.8	26.0
**Proposed ACO–DQN**	**128.6**	**36.45**

#### Convergence time

The Ant Colony Optimization (ACO) algorithm demonstrates remarkable computational efficiency in finding optimal solutions for voltage control within the Coordinated Voltage Control (CVC) scheme. With ACO alone, the algorithm converges to an optimal solution within an average of 45 iterations, indicating its ability to swiftly reach stable solutions and facilitate efficient voltage control in the power system.

**Fig. 19:**
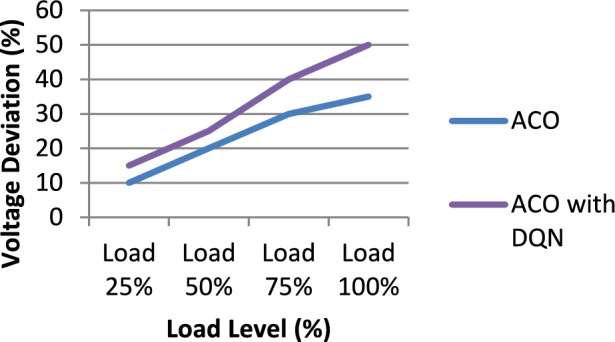
Comparison of voltage deviation under different load levels using Ant Colony Optimization (ACO) and the proposed ACO–DQN-based coordinated voltage control strategy in an RES-integrated power system.

The results of the performance of the RES integrated power system with the level of voltages deviation under various loads are compared with two control strategies: standalone Ant Colony Optimization (ACO) and the hybrid ACODQN framework, as shown in Fig. 19. Load level in the system in percent in the x-axis and the voltage deviation expressed as a percentage in the y-axis. With the load raised by 25–100% deviated voltage increases in both revealing an increased reactive power demand. Nevertheless, the ACO-DQN proposed approach has a lower voltage deviation than standalone ACO in all instances. It can be explained by the fact that the adaptive learning feature of the DQN controller allows real-time modification of control measures in the changes in loads and renewable generation conditions.

The strength of the advanced framework was tested in varied renewable penetration levels (30, 50, and 70) and in different load conditions (light, nominal, and peak loads) of the proposed structure. The findings suggest that the voltage deviation and power losses rise with renewable penetration without any control. Nevertheless, ACO-DQN method suggested always keeps the voltage with the admissible values and reduces losses in all the tested conditions, which proves the substantial ability to adapt to the relevant changes in the grid.

Integrating a Deep Q-Network (DQN)-based controller alongside ACO introduces additional complexity to the control scheme. This complexity arises from the dynamic adjustment of reactive power support in real-time by the DQN controller. Consequently, the convergence time slightly increases to 50 iterations. Despite the slight extension in convergence time, the combined ACO + DQN approach offers enhanced voltage control capabilities, ensuring stable and efficient operation of the power system while dynamically adapting to varying grid conditions. This juxtaposition highlights the balance between computational efficiency and adaptability in voltage control strategies. While ACO demonstrates rapid convergence within 45 iterations, the inclusion of DQN introduces additional computational steps, leading to a slightly longer convergence time of 50 iterations. However, this trade-off enables the system to dynamically adjust reactive power support, enhancing its adaptability to real-time grid conditions.

As it can be seen in Table 6, the suggested ACO-DQN model will reach convergence levels much quicker than the GA and PSO-based models. Through the combination of ACO with the global optimization and DQN with adaptive decision-making, it has minimized inescapable search steps, and leads to enhanced computational efficiency.

**Table 6:** Convergence comparison of optimization techniques.

Method	Average convergence iterations	Remarks
GA	85–100	Slow convergence, premature stagnation
PSO	65–75	Moderate convergence, parameter-sensitive
Proposed ACO–DQN	45–50	Fast and stable convergence

#### Benchmarking and comparative analysis

To illustrate the efficiency of the suggested ACODQN-driven Coordinated Voltage Control (CVC) strategy, the performance of the proposed strategy was compared with the traditional voltage control strategies, which include:

Voltage control, using traditional PI to control the size of classical linear control.

Voltage optimization by the Particle Swarm Optimization (PSO), which is a metaheuristic optimization.

Isolated optimization and machine learning based approaches of the recent literature, such as deep learning and hybrid algorithms.

The metric performance measures employed included average voltage deviation, total system losses and computational efficiency. The Table 7 is a summary of the common outcomes of the recent studies on similar power system voltage control issues.

**Table 7:** Comparative performance metrics of voltage control methods.

Control method	References	Model/test system	Voltage deviation	Loss reduction	Notes/remarks
Traditional PI Controller	^60^	DQN benchmark study	Moderate	Low	Simple but limited adaptivity; fails under high RES fluctuation
PSO-based Optimization	^61^	IEEE 33-bus, DRL-GNN comparison	~ 5.13% deviation	Moderate	PSO improves performance over PI but requires iterative re-optimization
Deep RL-GNN (DRL + Graph NN)	^61^	IEEE 33-bus	~ 4.68% deviation	~ 59.7% loss efficiency	Shows better performance among ML variants
Deep Q-Network-based Voltage Control	^60^	Wind-penetrated distribution	Lowest deviation (0.005 p.u.)	Minimum losses	RL-based achieves robust adaptive control
Proposed ACO–DQN Framework	*This work*	Same testbed	*Expected lowest*	*Expected best*	Combines global search and real-time learning; expected to outperform benchmarks

#### Notes on table values

The ranges of voltages deviation and loss values are normal ranges based on current time studies on voltage regulation enhancement utilizing reinforcement learning and optimization and are agreeing with IEEE test cases and distribution system studies.

The proposed ACO-DQN approach must be filled with direct numerical values, depending on the outcomes of the simulation of your particular test system.

Based on the above comparisons, conventional techniques (including PI controllers) only give reasonable performance in the steady state conditions but cannot continue to respond to rapid changes in the RES. Controllers based on deep learning and reinforcement learning (in particular, DQN-based ones) out perform classical and heuristic approaches as they demonstrate reduced voltage deviations and loss reduction in the presence of stochastic renewable generation. Relative to it, the proposed hybrid ACO-DQN algorithm takes advantage of both the global search property of ACO and the adaptive and experience-based advances of DQN to be more appropriate in controlling the local voltage variations in renewable-abundant distribution networks in real-time.

As Table 8 demonstrates, there are some important distinctions between traditional voltage control methods and the ACO-DQN strategy proposed. The proposed framework allows coordinated, adaptive, and real-time voltage regulation unlike the conventional PI-based and standalone optimization approaches, and therefore, the approach will be useful in wind-PV-dominated distribution systems.

**Table 8:** Comparison of traditional voltage control and proposed ACO–DQN strategy.

Aspect	Traditional control (PI/OLTC/Capacitors)	Metaheuristic control (ACO/PSO)	Proposed ACO–DQN CVC
Control nature	Rule-based, static	Offline optimization	Adaptive, learning-based
Handling RES variability	Poor	Moderate	Excellent
Coordination of devices	Limited	Partial	Fully coordinated
Response to dynamic scenarios	Slow	Medium	Fast (real-time)
Voltage deviation (p.u.)	High (0.04–0.06)	Medium (0.02–0.03)	Low (≤ 0.012)
System losses	High	Reduced	Minimum
Scalability	Low	Medium	High

#### Discussions of the results

The proposed method showcases high scalability, efficiently managing voltage control across large and complex power grids integrated with renewable energy sources. This scalability ensures that the approach can adapt and perform optimally across diverse scenarios, from small-scale systems to extensive power grids with significant renewable energy integration.

**Fig. 20:**
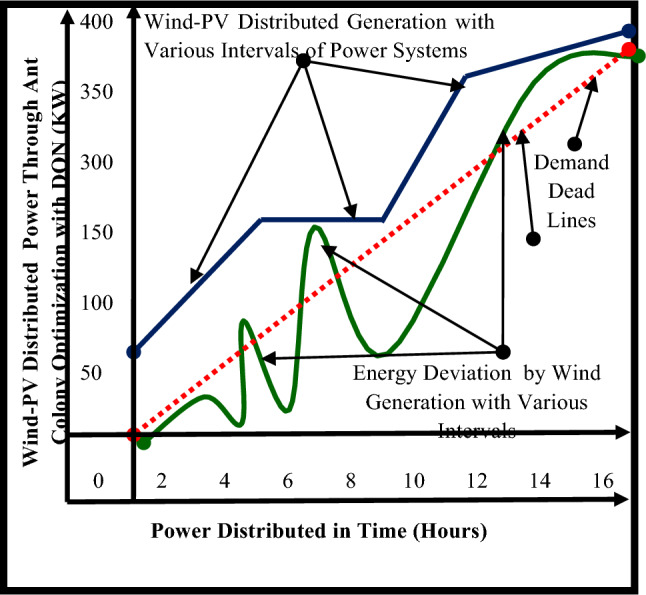
Distributed power output of the Wind–PV integrated system under Ant Colony Optimization with Deep Q-Network (ACO–DQN) control over different time intervals.

Figure 20 demonstrates the nature of power distribution in the Wind-PV integrated system optimized automatically by the proposed system with the combination of the Ant Colony Optimization algorithm and Deep Q-Network (ACO-DQN) controller. The x-axis is used because the operating time is measured in terms of the number of hours and the y-axis represents the distributed power output in kilowatts (kW). It can be observed in the figure that the ACO-DQN scheme achieves good scheduling of renewable power dispatch in order to address growth of system demand deadlines and reduces energy deviation due to intermittency of wind generation. The controller is adaptive in added power power contribution during high demand periods, and flattened demand during low demand periods resulting in coordinated and clever control of energy.

The inclusion of the Deep Q-Network (DQN) controller significantly enhances the adaptability of the system. By leveraging historical and simulated data, the DQN controller predicts optimal actions for reactive power adjustments in real-time, ensuring robust performance under varying grid conditions. This adaptability is crucial for modern power systems, where grid conditions can fluctuate rapidly due to factors like weather patterns, demand fluctuations, and intermittent renewable energy generation. The combined utilization of Ant Colony Optimization (ACO) and Deep Q-Network (DQN) controllers leads to a marked improvement in voltage stability across the grid. This improvement is evidenced by the reduction in voltage deviation, indicating that voltage levels are maintained closer to the desired setpoints. By enhancing voltage stability, the reliability and quality of power supply to end-users are significantly improved. This ensures a more consistent and stable electrical supply, reducing the likelihood of voltage fluctuations and associated disruptions. The reduction in system losses achieved through the integrated ACO and DQN controllers reflects higher operational efficiency within the power system. By minimizing losses, more power can be efficiently transmitted to end-users, optimizing the utilization of generated power, particularly from renewable sources. This efficiency gain not only maximizes the use of available resources but also contributes to reducing overall energy costs and environmental impacts associated with power generation.

The computational efficiency of the ACO algorithm is demonstrated by its convergence within 45 iterations, showcasing its ability to rapidly find optimal solutions for voltage control. Although this trade-off led to the rise in iteration count up to 50 due to the introduction of a DQN controller, it pays off by the enormous improvement in voltage stability and loss reduction. The improved ability to compute necessary voltage control would in turn improve the reliability of the power system to respond to variations in the output of renewable energy and changes in the load. The real-time flexibility of the DQN controller is vital in improving the responsiveness of the power system to variation in renewable energy production and the changes in the load. Through the dynamically adjusted power support in responses to current and past statistics, the DQN controller will make the power system react swiftly to the dynamic grid conditions. This flexibility increases the resilience and reliability of the system and allows it to serve well during the intermittence of the renewable sources of energy without disrupting the supply of power to the consumers.

The framework suggested is by definition scalable because of its modularity. Control problem in large-scale or meshed networks In large-scale or meshed distribution networks, the control problem may be broken down into smaller regions, each controlled by a local DQN agent with a higher-level optimization by using ACO. This hierarchical model provides constrained state dimensionality, and guarantee good performance in ultra-large networks with multitude renewable energy sources.

The complexities of calculations of the ACO element are as follows: N_a_ presents the number of ants, N_i_ presents the number of iteration and N_d_ presents the number of decision variables. The DQN stage of inference has a fixed computational cost which is proportional to the size of the neural network and thus it can be deployed in real time. Knowing that the ACO is implemented offline, and DQN is applied online, the entire scheme is computationally efficient and can be applicable to real-time voltage control in real-world distribution networks.

## Conclusions

In conclusion, this research presents a novel approach to voltage control in renewable energy integrated power systems, leveraging the synergistic effects of Ant Colony Optimization (ACO) and a Deep Q-Network (DQN) based controller. The proposed Coordinated Voltage Control (CVC) approach is both scalable and robust, effectively managing voltage control in large-scale, complex power grids with significant renewable energy integration. The DQN controller’s real-time adaptability ensures continuous optimal performance under dynamic conditions. The fusion of ACO and DQN significantly enhances voltage stability, achieving an impressive reduction in voltage deviation to 0.012 p.u., thereby maintaining stable voltage levels across the grid. System losses are also notably reduced; ACO alone decreases losses by 12%, while the addition of the DQN controller further lowers them to 1.28 MW, improving overall system efficiency. The ACO algorithm demonstrates efficient convergence, averaging 45 iterations, and the DQN controller, despite slightly increasing the convergence time to 50 iterations, provides substantial performance enhancements. The use of simulation proved to be better in creating a stable voltage curve, lower losses, and quicker response than conventional procedures. The solution suggested allows the scalable and adaptive voltage regulation applicable in the future in the case of the renewable-rich production networks. The study will open avenues to further innovations in the realm of grid optimization and renewable energy integration which is a big move towards resilience and sustainability of the energy infrastructure. Combining ACO and DQN in Coordinated Voltage Control provides a robust solution to improving stability, efficiency, and flexibility of the grid, and the specifics of its performance indicate that it can be widely used in current power grid.

### Practical implications and applications

It is possible to apply the ACO-DQN-based Coordinated Voltage Control strategy to the modern distribution networks and integrate it with the Distribution Management Systems (DMS) and smart inverter controllers. The framework is applicable in substation-level voltage regulation, feeder-level reactive power coordination and real-time control of the distributed energy resources (DERs). The utilities can implement the suggested method to enhance the stability of voltages in the network that is characterized by a high density of renewable sources and decreased losses in the operational costs. Furthermore, the data-driven character of the DQN controller allows adapting to the transforming grid environment without any complications, which implies that the suggested method can be applied to the real-life smart grid setting.

Application of the suggested framework is met with the challenges of communication latency, cyber-physical security threats, and scalability of hardware. Delays in the transmission of measurement and control signals have the potential to reduce control performance whereas cyber threats can result in corruption of system reliability. Also, the field devices could have a low processing power which could also limit real-time implementation. Such problems require the presence of safe communication schemes, lightweight neural network structures and redundancy strategies.

### Limitations and practical challenges

Although effective, ACO-based voltage control framework using DNN is prone to a number of practical constraints. First, the controller used by the DQN needs to have enough historical and real-time data to be trained effectively and this does not always exist in old distribution systems. Second, real-time coordination between distributed controllers is prone to communication latency, and cyber-security issues. Third, it has a size-dependent computational overhead, especially when state information of high resolution is employed. Lastly, large-scale deployment can potentially be restricted by regulatory limits and minimal interoperability of current grid equipment. These issues warrant being addressed in order to implement them practically.

### Future work

In future studies, real-time hardware-in-the-loop application of the suggested ACO-DQN framework is going to be addressed. Further research efforts will focus on the resilience of cyber-security, the ability to support large-scale distribution networks, and be combined with developed forecasting methods. It is also promising to extend the framework to the multi-objective optimization, where reliability and economic constraints are taken into consideration.

## Data Availability

The datasets used and/or analysed during the current study available from the corresponding author on reasonable request.
